# Defining neuroblastoma: From origin to precision medicine

**DOI:** 10.1093/neuonc/noae152

**Published:** 2024-08-05

**Authors:** Lourdes Sainero-Alcolado, Tomas Sjöberg Bexelius, Giuseppe Santopolo, Ye Yuan, Judit Liaño-Pons, Marie Arsenian-Henriksson

**Affiliations:** Department of Microbiology, Tumor and Cell Biology (MTC), Biomedicum B7, Karolinska Institutet, Stockholm SE-17165, Sweden; Department of Women’s and Children’s Health, Karolinska Institutet, Stockholm SE-17177, Sweden; Paediatric Oncology Unit, Astrid Lindgren’s Children Hospital, Solna SE-17164, Sweden; Department of Microbiology, Tumor and Cell Biology (MTC), Biomedicum B7, Karolinska Institutet, Stockholm SE-17165, Sweden; Department of Microbiology, Tumor and Cell Biology (MTC), Biomedicum B7, Karolinska Institutet, Stockholm SE-17165, Sweden; Department of Microbiology, Tumor and Cell Biology (MTC), Biomedicum B7, Karolinska Institutet, Stockholm SE-17165, Sweden; Department of Laboratory Medicine, Division of Translational Cancer Research, Lund University, Lund SE-22381, Sweden; Department of Microbiology, Tumor and Cell Biology (MTC), Biomedicum B7, Karolinska Institutet, Stockholm SE-17165, Sweden

**Keywords:** immunotherapy, neural differentiation, neuroblastoma, sympathoadrenal development, targeted therapies

## Abstract

Neuroblastoma (NB), a heterogenous pediatric tumor of the sympathetic nervous system, is the most common and deadly extracranial solid malignancy diagnosed in infants. Numerous efforts have been invested in understanding its origin and in development of novel curative targeted therapies. Here, we summarize the recent advances in the identification of the cell of origin and the genetic alterations occurring during development that contribute to NB. We discuss current treatment regimens, present and future directions for the identification of novel therapeutic metabolic targets, differentiation agents, as well as personalized combinatory therapies as potential approaches for improving the survival and quality of life of children with NB.

## Graphical Abstract

**Graphical Abstract:**
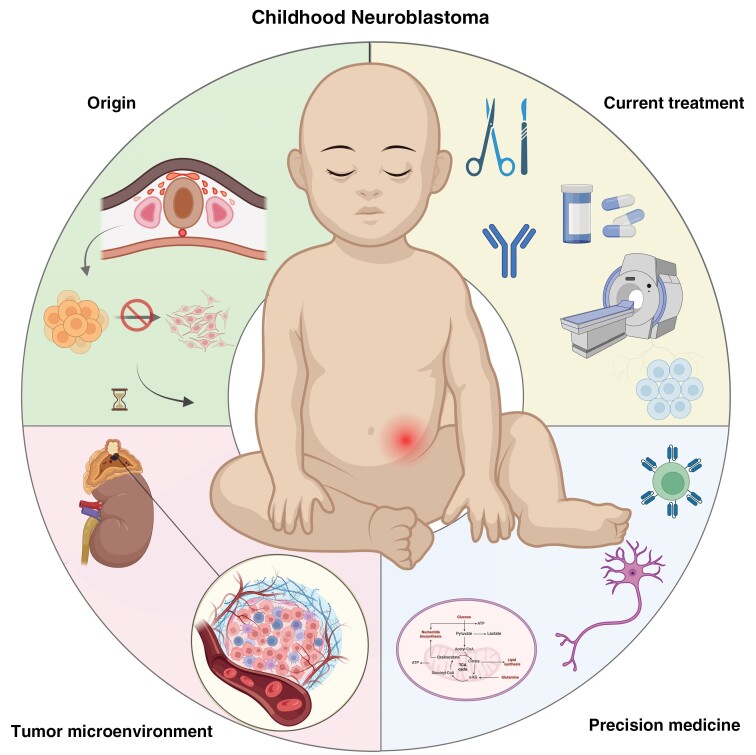
Neuroblastoma is the most common extracranial solid tumor of childhood, and significant progress has been achieved in understanding its origin, biology, and new, more efficient therapies. In this review, we discuss the latest advances in defining the cell of origin, the current treatment protocols, and the precision medicine strategies under development, including metabolism, induction of differentiation, and immunotherapy, as well as the implications of the tumor microenvironment on tumorigenesis and as a treatment target.

Children rarely develop cancer, yet the incidence of pediatric tumors has increased during recent years, emerging as one of the leading causes of death during childhood.^1^ The most common tumors in children include leukemias, lymphomas, neuroblastoma (NB), retinoblastoma, bone tumors, sarcomas, and tumors affecting the central nervous system [CNS (medulloblastoma, ependymoma, and glioma)].^2^ In contrast to adult cancer, tumors in children have a very low mutational rate, and only approximately 10% show epigenetic alterations.^3^ Most of these gene alterations occur prenatally in different immature cell populations. Neuroblastoma, accounting for 7% of all cancers in childhood (nearly 13 per million cases), is the most common solid extracranial pediatric cancer that emerges during the first year of life. It stems from neural crest-derived precursors of the sympathetic nervous system, mainly manifesting in the adrenal gland and paraspinal ganglia. The cancer is heterogeneous, ranging from tumors that regress spontaneously, to others that metastasize and are therapy resistant.^4,5^ Occasionally, NB patients have other neural crest-derived diseases, including congenital central hypoventilation syndrome, Hirschsprung disease, neurofibromatosis type 1, or phaeochromocytoma,^6^ suggesting a common role of specific genes in their development (Supplementary Table S1).

Current treatments depend on the age of patients and the stage of the disease. The most aggressive protocol is administered to children affected with high-risk NB, characterized by undifferentiated tumors with poor prognosis. Despite this regimen, only 50% of patients are cured^7^ and most of these children suffer from long-term side effects impacting their development and quality of life.^8^ Thus, new strategies, such as personalized therapies and follow-up guidelines, are urgently needed to ameliorate these adverse effects for survivors. Here, we discuss the latest studies on promising new therapeutic targets, including novel small molecule compounds inducing differentiation or impacting metabolism, and recent advances in immunotherapy, as well as different laboratory models in NB research.

## Origin of Neuroblastoma

Although NB originates during development, the exact timing and the cell or cells of origin are still under debate. Following the closure of the neural tube, neural crest cells (NCCs) detaching from its dorsal side undergo a transition from an epithelial to a mesenchymal phenotype, migrating towards the side of the aorta, and differentiating into several cell types. The anteroposterior and dorsoventral identity of the NCCs is controlled by gradients of growth factors and morphogens, including Wingless and Int-1 (WNT), bone morphogenic protein (BMP), retinoic acid (RA), and fibroblast growth factor (FGF).^9^

**Figure 1.**
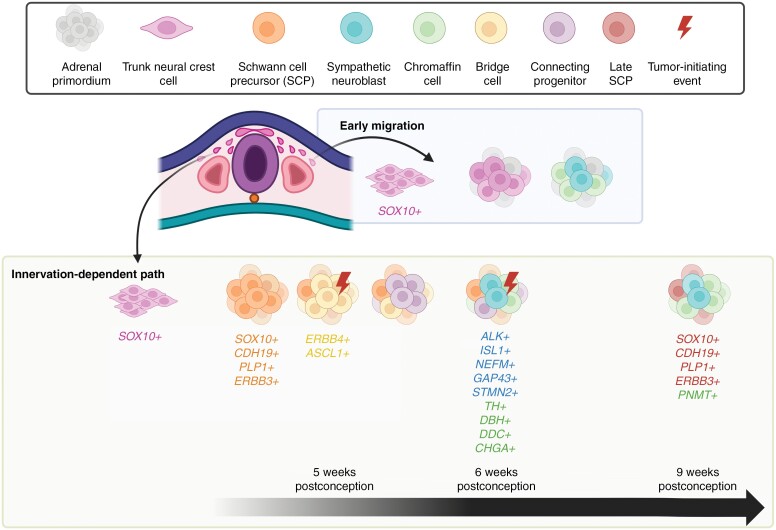
Cell types involved in the development of the adrenal gland and proposed NB triggering events. Upper panel: definition of the cells during adrenal gland development in color code as indicated. Lower panel: early migration and the innervation-dependent path events, highlighting the cell types involved and their specific gene expression indicated by the corresponding color code. During the early migration, neural crest cells, migrate near the region of the dorsal aorta and can differentiate directly into sympathetic neuroblasts and chromaffin cells. In the innervation-dependent path, neural crest cells differentiate into cycling SCPs. These precursors can then progress through an intermediate stage, known as bridge cells, and further into connecting progenitor stage. These will then differentiate into sympathoadrenal neuroblasts, which can also become chromaffin cells. The remaining SCPs will differentiate into late SCPs. The potential stages for tumor-initiating events are indicated. *CDH19*: Cadherin 19; *PLP1*: proteolipid protein; *ERBB3/4*: *erb-b2* receptor tyrosine kinase 3/4; *ASCL1*: achaete-scute family bHLH transcription factor 1; *ISL1*: ISL LIM homeobox 1; *NEFM*: neurofilament medium chain; *GAP43*: growth associated protein 43; *STMN2*: stathmin 2; *TH*: tyrosine hydroxylase; *DDC*: dopa decarboxylase; *CHGA*: chromogranin A; *PNMT*: phenylethanolamine N-methyltransferase.

The progress of novel molecular tools with single-cell resolution has provided deeper insights into the development of the peripheral nervous system and NB formation, challenging previous models (Table 1). A novel population of Schwann cell precursor (SCP)-derived cells in the developing adrenal gland, called bridge cells, has been reported to generate chromaffin cells and then disappear during organ maturation.^20^ Using single-cell RNA-sequencing (scRNA-seq) of the developing human adrenal gland, Jansky et al. identified NCC-derived SCPs as the origin of both adrenal chromaffin cells as well as sympathoadrenal neuroblasts.^12^ According to the differentiation model generated, trunk NCC (tNCC)-derived SCPs differentiate, via the bridge cells, into the newly described connecting progenitor cells, which then can further differentiate into both chromaffin cells as well as sympathetic neuroblasts^12^ (Figure 1). Based on RNA velocity studies, sympathetic neuroblasts differentiate into chromaffin cells in the developing human adrenal gland,^14^ where maturation continues until three years of age. This is evidenced by the presence of a population of chromaffin-like progenitor cells identified in the postnatal human adrenal gland.^11^

**Table 1.** Single-cell RNA-sequencing studies of normal human adrenal glands and tumors. Reference to the study, number and type of samples, sequencing technology platform, and main conclusions are specified

Study	Samples type	INSS/INRGSS	*N*	Platform	Main conclusions
Kildisiute et al., 2021^10^	Adrenal gland (8 to 21 wpc)		7	10× genomics	Neuroblastoma cancer cell resembled fetal sympathoblasts, but no other fetal adrenal cell type.
	Neuroblastoma	2A	2	10× genomics; CEL-Seq2	
		3	3		
		4	15		
		NA	1		
Bedoya-Reina et al., 2021^11^	Adrenal gland (adult)		3	SMART-seq2	Low-risk neuroblastoma resembled sympatho- and chromaffin cells, while high-risk neuroblastoma resembles a subtype of *a TRKB* + cholinergic progenitor population.
	Neuroblastoma	1	1		
		2A	1		
		2B	1		
		3	2		
		4	4		
		4S	2		
Jansky et al., 2021^12^	Adrenal gland (7 to 17 wpc)		17	10× genomics	Schwann cell precursors differentiate via intermediate states to neuroblasts or chromaffin cells; Neuroblastomas resemble normal fetal adrenal neuroblasts.
	Neuroblastoma	NA	14		
Dong et al., 2020^13^	Human embryo (4 wpc)		2	10× genomics	Most of the tumor cells resemble developing chromaffin cells with fewer sympathoblasts (different assignment of chromaffin and sympathoblast markers).
	Adrenal gland (8 to 14 wpc)		4		
	Neuroblastoma	NA	14		
	Ganglioneuroblastoma		2		
Kameneva et al., 2021^14^	Adrenal gland (6 to 14 wpc)		11	10× genomics	Intra-adrenal sympathoblasts come from Schwann cell precursors, while most extra-adrenal sympathoblasts are from the migratory neural crest.
	Neuroblastoma (Olsen et al. 2020)	NA	2		
	Neuroblastoma (Dong et al. 2020)	NA	6		
Verhoeven et al., 2022^15^	Neuroblastoma	L1	2	10× genomics	The immune landscape of NB presents 27 different immune cell subtypes.
		L2	6		
		M	10		
		NA	1		
Costa et al., 2022^16^	Neuroblastoma	Localized	1	10× genomics	NB tumors have an immunocompromised microenvironment characterized by dysfunctional T cells and accumulation of immunosuppressive cells.
		M	1		
		3	1		
		4	4		
		4S	3		
Yuan et al., 2022^17^	Neuroblastoma	3	2	Smart-seq2	High tumor heterogeneity and plasticity between adrenergic and mesenchymal cell states via an intermediate state termed “transitional.”
		4	3		
	Ganglioneuroma		1		
	Ganglioneuroblastoma		2		
Liu et al., 2022^18^	Peritumoral adrenal gland		3	Gexscope	C1 subgroup of malignant tumor cells may differentiate into a C2 subcluster of fibroblasts, revealing a novel mechanism of spontaneous NB regression.
	Neuroblastoma	1	2		
		1 to 2	1		
		2	4		
		2B	1		
		4	5		
		4S	4		
Thirant et al., 2023^19^	Neuroblastoma	Localized	4	10× genomics	NB tumors present intratumor heterogeneity and cellular plasticity between mesenchymal and noradrenergic identities.
		3	1		
		4	10		
		4S	3		

While an earlier study from Dong et al. identified a chromaffin phenotype for NB (reporting chromaffin differentiation state as a prognostic factor^13^), this interpretation has been attributed to a reversed manual annotation of cell types between mouse and human.^12,14,10^ Both Jansky et al. and Kameneva et al. reported transcriptional similarities between the developing sympathoadrenal neuroblasts and tumor samples from patients. Notably, low-risk NB was reminiscent of committed neuroblasts, while *MYCN*-amplified tumors had strong mesenchymal features and resembled bridge cells and early neuroblasts.^12,14^ These results indicate the potential of neuroblasts as the cell of origin. Moreover, undifferentiated cells from high-risk NB were described by Bedoya-Reina et al. to bear a strong resemblance to the progenitor population identified in the postnatal adrenal gland.^11^ In contrast, low-risk NB resembled postnatal chromaffin cells as well as more committed fetal neuroblasts and chromaffin cells. The authors also showed that high-risk NB had transcriptomic features associated with late disease development, while low-risk cases correlated with younger age at diagnosis. This suggests that NB may arise from different cell types at specific developmental stages, explaining the high heterogeneity observed between patients. High-risk NB may be the result of either fast-cycling progenitor cells that undergo tumorigenic transformation during development, or cells that, due to mutation or chromosomic aberration, fail to initiate differentiation postnatally. Low-risk NB on the other hand might be generated by cells that maintain a certain proliferative capability but are more committed towards differentiation and have a lower rate of cell division. Notably, NB cells have the ability to reprogram, ie, to switch between different cellular states. For instance, the overexpression of the paired-related homeobox 1 (PRRX1) transcription factor can forcibly change the state of NB cells in vitro from adrenergic to mesenchymal.^21^ This plasticity and epigenetic control enable them to adapt to various microenvironmental contexts contributing to shaping tumor heterogeneity.

Körber et al. investigated the timing and effect of genetic events for NB development and prognosis.^22^ They reported that chromosomal aberrations leading to disease development occur within the first trimester of pregnancy in the majority of NB cases analyzed. Moreover, they observed that tumors with a longer evolutionary history were associated with more aggressive forms of malignancy. In line, Gundem et al. conducted clonal tracing and showed as expected that NBs originate during embryogenesis, and also found that dormant metastatic clones were already present at diagnosis. Interestingly, single nucleotide variants at disease-defining loci (ie, *Alpha-thalassemia/mental retardation, X-linked* (*ATRX*) and*Telomerase reverse transcriptase* (*TERT*)) increased with time in the trunk, this area defined as the most recent common ancestor as identified by clonal tracing.^23^

Taken together, these data suggest that several types of progenitor cells emerge during adrenal gland development and contribute to the formation of the distinct cell types composing this organ, to then exhaust their stem-like properties and terminally differentiate. Neuroblastoma could directly arise from these progenitors, or from cell populations still capable of reactivating developmental programs similar to those of the adrenal gland. The phenotypical resemblance with the different progenitor populations may be the cause behind the heterogeneity and properties of the different stages of NB.

## Diagnosis, Disease Stages, and Prognosis

Diagnosis is based on clinical findings characteristic of NB: age at onset, presence of abdominal mass combined with radiological findings suggesting NB, and pending biomarkers such as urinary catecholamines. Histopathological exam of the biopsy confirms the final diagnosis. Prognosis involves several factors such as age at diagnosis, tumor location, and genetic testing to identify NB-associated segmental chromosomal aberrations and mutations, especially in *MYCN* and *Anaplastic Lymphoma Kinase* (*ALK)*.

The heterogeneity of NB has hampered classification and treatment options, and thus phenotypes have been grouped into different categories. A first attempt at creating a more comprehensive system resulted in the International Neuroblastoma Staging System (INSS),^24^ which was predominatly used for several decades (Supplementary Table S2).

In recent years, additional factors determining NB treatment and risk of relapse have been identified, such as chromosomal rearrangements, including 1p and 11q loss and 17q gain,^25–27^ and histological feature assessment.^28^ For this purpose, the International Neuroblastoma Risk Group (INRG) developed the INRG staging system (INRGSS),^29^ which stratifies patients in 16 pretreatment groups lettered A through R. Each group is associated with one of the four risk categories: very low risk, low risk, intermediate risk, or high risk (Supplementary Table S3).

During staging and risk assessment, particular importance is given to the status of *MYCN* (amplified *versus* non-amplified). MYCN is one of the most important regulators of cell proliferation and its amplification is associated with more aggressive tumor types, which makes it the most important stratification factor while evaluating NB risk groups. Tumor histopathology (favorable *versus* unfavorable) is another important element of risk stratification, which can distinguish NB from the less aggressive ganglioneuroblastoma or ganglioneuroma. Neuroblastoma is divided into three histological subtypes: undifferentiated (always unfavorable), poorly differentiated, and differentiated, where the latter usually is favorable, depending on the presence and level of the mitosis–karyorrhexis index.^30^

Notably, some NB cases appear confined to the primary site and only spread to the skin, liver, and/or bone marrow in infants younger than 12 months. The bone marrow is usually involved in less than 10% of cases examined. In the INSS classification, these tumors are referred to as stage 4S, equivalent to INRGSS MS. For these patients, clinicians can adopt a treatment-free/observation strategy as these tumors very often undergo spontaneous remission^31^ and, in case of tumor growth, surgery with or without chemotherapy is sufficient for complete recovery.^32^ Patients with asymptomatic stage 4S/MS without hepatomegaly and with non-*MYCN*-amplified tumors, hyperdiploid, and favorable histology are followed during the initial stages. Up to 70% of these patients show spontaneous regression and do not require treatment.^33^

## Spontaneous and Familial Genetic Alterations

Familial NB is a rare form of the disease and only occurs in 1–2% of all cases, caused by inherited genetic mutations that increase the risk of cancer development.^34^ Sporadic NB, on the other hand, is the most common form and occurs in individuals without a family history. It is widely postulated that multiple germline variations synergistically contribute to an increased likelihood of disease development.

Specific genetic alterations associated with NB include *MYCN* amplification, mutations in the *ALK*, *Paired-like Homeobox 2B* (*PHOX2B*), and *ATRX* genes. Additionally, *TERT* rearrangements are common.^35,36^ The proteins encoded by these genes play critical roles in tumor development and progression and are being explored as potential targets for new therapies.

### *v-myc Avian Myelocytomatosis Viral Oncogene NB-Derived Homolog* (*MYCN*)

Amplification of *MYCN* occurs in about 20% of all NB cases while in 40% of the high-risk group patients, and is a strong marker of high risk and poor prognosis,^37^ associated with resistance to chemotherapy and radiotherapy. Notably, *MYCN* mutations are uncommon and only account for 1.7% of the cases.^38^

MYCN belongs to a family of basic helix-loop-helix-leucine zipper (bHLH-Zip) transcription factors, together with c-MYC and MYCL. The MYC proteins form heterodimers with MAX and activate or repress transcription of target genes, regulating key cellular processes including cell cycle, ribosome biogenesis, cell growth, apoptosis, and metabolism.^39^ High expression of MYC/MYCN target genes is associated with poor survival independently of *MYCN* status, age at diagnosis, or disease stage.^39^

The amplification of *MYCN* contributes to the development and progression of NB by promoting cell growth and survival, as well as by inhibiting cell death.^40^ In addition, MYCN contributes to the maintenance of an undifferentiated phenotype and to metabolic reprogramming. In transgenic mice, *MYCN* expression in migrating NCCs or sympathetic progenitors under the control of the rat tyrosine hydroxylase (*TH*) promoter results in NB development resembling human tumors with *MYCN* amplification.^40^ Transgenic *MYCN* expression in additional mouse or zebrafish models also recapitulates NB tumorigenesis, proving its role as an oncogenic driver (Supplementary Table S4).

Apart from *MYCN* amplification, both *MYC* and *MYCN* are found upregulated in NB, either by amplification of enhancer elements or by chromosomal translocations leading to enhancer hijacking, but never together.^41^ Interestingly, several missense mutations of *MYCN* and *MYCN opposite strand* (*MYCNOS*) have also been observed in patients with NB (St Jude PeCan database).^42^

Currently, there is no treatment for targeting MYCN or MYC in cancer. However, the potential of personalized therapies that affect *MYC* transcription, protein stability, dimerization with MAX, or its cofactors has been studied intensively.^43^ The most promising approach to date is the 91 amino acid-long Omomyc peptide that preferentially binds to MAX, but also forms homodimers, and heterodimers with MYC, thus impairing MYC-driven transcription. This peptide recently passed a phase I clinical trial for advanced adult solid tumors, showing a favorable safety profile and stable disease in some of the patients.^44^

### Anaplastic Lymphoma Kinase (ALK)

Approximately 5–10% of NB patients have mutations in *ALK*, observed both in sporadic as well as in familial cases, suggesting its essential role in tumorigenesis. During physiological conditions, *ALK* is expressed in stem and progenitor cells during development, and particularly in NCCs.^45^ ALK has also been reported to induce expression of the receptor tyrosine kinase REarranged during Transfection (RET) as well as its phosphorylation, required for sympathetic neuron development.^46^

The most frequent gain-of-function germline mutations of ALK in familiar NB are G1128A, R1192P, and R1275Q.^47^*ALK* also plays an important role in relapse, with up to 15% of cases acquiring activating mutations. Drugs including ceritinib, alectinib, and brigatinib have been developed to treat cancers with mutations in the *ALK* gene, including lung cancer and NB, demonstrating a higher efficacy than older drugs like crizotinib, especially against specific *ALK* mutations. Combining ceritinib with inhibitors of the tyrosine kinase receptor AXL or the cyclin D1/CDK4 and CDK6 inhibitor ribociclib can improve its effectiveness, while alectinib and brigatinib have also shown promise in treating NB with *ALK* mutations.^48^ Preliminary results from the ongoing phase I clinical trial for the ALK and ROS1 third-generation inhibitor lorlatinib showed both safety and efficacy in pediatric, adolescent, as well as in adult NB patients.^49^ In addition, a study of the genetic alterations in *ALK* in both primary and relapsed cases of NB showed that the de novo *ALK* point mutation R125Q is more frequent upon relapse. This suggests the relevance of studying the genomic *ALK* status in all NB cases that relapse or in which the disease progresses, to assess their sensitivity to ALK inhibitory treatment. This work also revealed high frequencies of *ALK* mutations in intermediate-risk NBs of young stage 4 patients, suggesting the potential therapeutic benefits of ALK inhibitors in this subgroup.^50^ Point mutations and plasticity of NB are key factors in determining the response to ALK inhibition when compared to the adult inflammatory myofibroblastic tumors or non-small cell lung carcinomas, which are more sensitive and present instead *ALK* fusion proteins.

### Paired-Like Homeobox 2B (PHOX2B)

Genetic alterations in *PHOX2B* were the first associated with increased susceptibility for NB. In fact, mutations in the *PHOX2B* gene are found in about 6% of NB cases, and high levels of *PHOX2B* have been associated with poor prognosis in patients with high-risk disease.^51^

The *PHOX2B* gene is expressed by sympathetic neuroblasts and SCPs, and regulates growth and differentiation of sympathoadrenal progenitors, specifically during the transition between neuronal progenitors and noradrenergic neuron populations.^52^ Recent studies have shown that *PHOX2B* is expressed in a subset of NB cells with adrenergic properties, and its knockdown impairs the growth of these cells, as evidenced by mapping the NB super-enhancer landscape.^21^

### Other Genetic Alterations

The *ATRX* gene is involved in the development of the nervous system as well as the regulation of neuronal differentiation and encodes a SWItch/Sucrose Non-Fermentable (SWI-SNF)-like chromatin remodeling protein. Mutations of *ATRX* are associated with all adolescent and young adults (>12 years old) NB cases, but germline mutations do not increase susceptibility to the disease. *ATRX* mutations are also observed in 17% of children between 18 months and 12 years of age.^53^ They seem to have a tumorigenic effect through a loss-of-function mechanism, and mutations appear to affect histone H3.3 chaperone function, leading to genome instability and silencing of genes involved in neuronal differentiation, including the *retinoic acid receptor alpha* (*RARA*) gene. Importantly, loss-of-function of *ATRX* confers sensitivity to poly-ADP ribose polymerase (PARP) inhibitors.^54^ In contrast to *ALK*, *ATRX* mutations never occur together with *MYCN-*amplification^55^ as they lead to the alternating lengthening of telomeres (ALT) phenotype, which is mutually exclusive with *TERT* rearrangements involved in telomerase activation induced by MYCN.^56^*MYCN* amplification induces metabolic reprogramming, mitochondrial dysfunction, and oxidative and DNA-replication stress, and *ATRX* mutations cause additional replicative stress through defects in the ATRX-histone chaperone complex. The combination of replicative stress caused by ATRX and MYCN alterations causes synthetic lethality in NB cells.^55^

Furthermore, alterations in the p53 tumor suppressor and the TERT proteins are observed in tumors of certain NB patients. Despite rarely mutating in NB, *TP53* expression levels are directly correlated to *MYCN* amplification and high expression is a negative prognostic factor.^57^ Tumors carrying *TERT* rearrangements or *MYCN* amplification result in high *TERT* expression levels and are associated with an unfavorable prognosis.^58^*Forkhead box R2* (*FOXR2*), encoding the FOXR2 transcription factor, is aberrantly upregulated in 70% of human tumors, while only in 9% of NB patients. This protein stabilizes MYCN, resulting in very similar outcomes as *MYCN*-amplified cases.^59,60^ Additional genes, encoding chromatin remodeling factors, have also been involved in NB development including chromodomain helicase DNNA-binding protein (*CHD5*), AT-Rich Interaction Domain 1A/1B (*ARID1A*/*ARID1B*), Brahma related gene 1 (*BRG1*), and DNA methyltransferase 3 alpha/beta (*DNMT3A/B*).^61^

## Neuroblastoma Therapy

Treatment stratification and risk group assignment for NB varies based on tumor stage and cytogenetics. For high-risk (INSS stages 2 to 4, and 4S with *MYCN* amplification, or INSS stage 4 without *MYCN* amplification > 12 months at diagnosis) or stage 4/M patients, a multimodal therapy strategy is employed, including surgery, radiotherapy, chemotherapy, autologous stem-cell transplantation, and immunotherapy. For certain low-risk cases, a watch-and-wait approach is adopted, with surgery being sufficient for complete remission upon tumor growth.^62^ In the case of high-risk patients with metastatic disease, the benefits of macroscopic surgical resection of the primary tumor are still under debate.^63^

The frontline or standard of care treatment protocols for high-risk NB includes several phases.

### Induction Phase

Multiagent chemotherapy is administered to reduce tumor size locally and at metastatic sites prior to surgery and radiotherapy. In the US, the most common induction regimen according to Children’s Oncology Group (COG) includes five cycles of intensive chemotherapy with a combination of vincristine, cyclophosphamide, topotecan, doxorubicin, cisplatin, and etoposide,^64^ while some sites have different protocols, for instance the Memorial Sloan Kettering Cancer Center (MSKCC).^65^ The SIOPEN protocol used in Europe consists of cisplatin, vincristine, carboplatin, etoposide, and cyclophosphamide, known as rapid COJEC.^66^

### Local Treatment Phase

This step comprises surgery and radiotherapy with the aim to perform such a complete macroscopic resection as possible, without causing excessive morbidity, and is usually performed at the end of induction phase. To reduce risk of local relapse, radiotherapy is offered to site of primary tumor and given after clinical and hematological recovery after HDC/ASCR or myeloablative therapy during the consolidation phase.

### Consolidation Phase

To sustain and deepen remission with high-dose chemotherapy with autologous hematopoietic stem-cell rescue (HDC/ASCR). To enter this phase, a sufficient tumor response needs to be achieved including at least partial response in metastatic sites. Tandem autologous hematopoietic stem-cell transplantation has been shown to improve disease outcome as a consolidation approach in patients with high-risk disease^67^ and is now incorporated into an ongoing clinical trial (NCT04221035). Primary site radiotherapy is included in the COG and SIOPEN protocols, but its use in metastatic lesions is controversial.^68^

### Maintenance Therapy

This step targets minimal residual disease to prevent relapse. Patients receive several courses with an anti-GD2 (disialoganglioside) monoclonal antibody (dinutuximab beta in Europe or naxitamabin in the US) combined with isotretinoin, inducing cell differentiation in some patients although reliable biomarkers to predict response are absent.^69^

Palliative radiotherapy is recommended when life- or organ-threatening complications are expected, or upon disease progression despite surgery and chemotherapy.^70^

## Neuroblastoma Microenvironment

The NB tumor microenvironment (NB-TME) consists of the extracellular matrix (ECM), stromal, endothelial, and immune cells, which interact with cancer cells and release growth factors, cytokines, and chemokines, that in turn affect cancer cell behavior and tumor progression.^71^ Understanding the NB-TME composition is key for precision medicine, as it can affect treatment response.

The ECM components play a critical role in NB-TME where collagen and fibronectin provide structural support to the tumor and can modulate the behavior of cancer cells. For example, collagen IV alters the expression of integrins thus modulating NB cell adhesion, migration, and invasion.^72^ Indeed, ECM composition, including blood vessel organization and reticulin fibers, can delineate an ultra-high-risk NB subgroup with a survival rate below 15%.^73^

**Figure 2.**
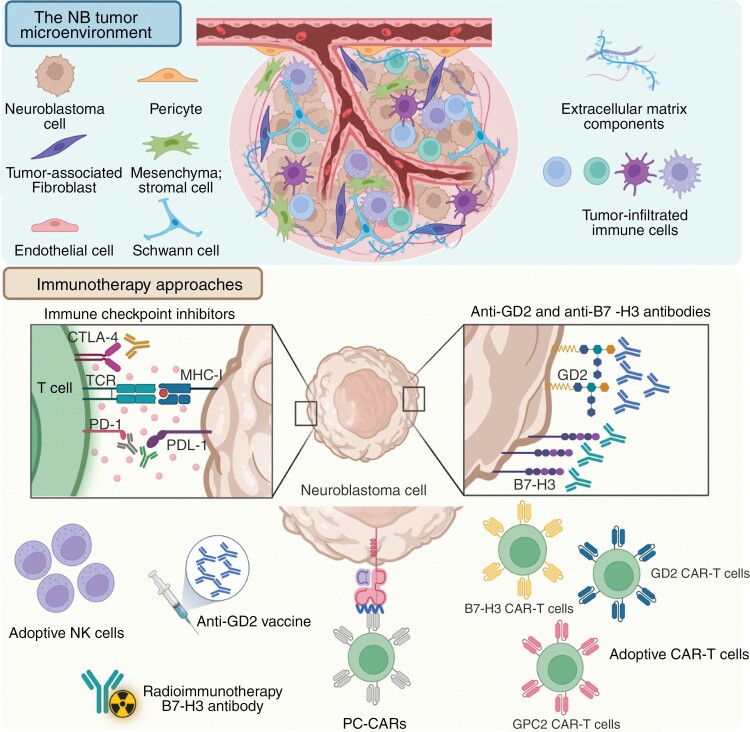
The NB tumor microenvironment and current immunotherapy approaches. Graphical illustration of the different components of the NB tumor microenvironment (upper panel) and the immune-based approaches currently implemented or explored as NB therapies (lower panel). This figure represents the complex interplay of the heterogenous cellular and molecular components within the NB tumor microenvironment: tumor cells, stromal cells (pericytes, tumor-associated fibroblasts, endothelial and mesenchymal stromal cells), immune cells (tumor-associated macrophages, dendritic cells, NK cells, B and T lymphocytes), SCs, and extracellular matrix (collagen, fibronectin, and laminin). The available immunotherapies for NB treatment include immune checkpoint inhibitors such as anti-CTLA-4, anti-PD-1/PDL-1, anti-GD2 and anti-B7-H3 mAbs, and adoptive NK cells, radioimmunotherapy with B7-H3 mAbs, anti-GD2 vaccine, PC-CARs, and CAR-T cells. CTLA-4: cytotoxic T-lymphocyte-associated protein 4; TCR: T-cell receptor; PD-1: programmed cell death protein 1; PDL-1: programmed cell death ligand 1; MHC-I: major histocompatibility complex class I; GD2: disialoganglioside; CAR-T: chimeric antigen receptor T-cell; PC-CARs: peptide-centric chimeric antigen receptors; NK: natural killer cells.

Cells in the NB-TME can be divided into immune, including tumor-associated macrophages (TAMs), dendritic cells, natural killer (NK), natural killer T (NKT) cells, B and T lymphocytes, and non-immune cells comprising endothelial cells, pericytes, Schwann cells (SCs), mesenchymal stromal cells, and cancer-associated fibroblasts (CAFs). Depending on phenotype and status, immune cells suppress or promote tumor growth (Figure 2). *MYCN*-amplified tumors are generally considered “cold,” with low immune cell infiltration, while the non-*MYCN*-amplified are regarded as “hot,” containing several types of immune cells (B, NK, NKT cells, M2 macrophages).^74^ Single-cell RNA-sequencing technologies defined the NB immune landscape, with 27 different immune subtypes, dysfunctional T cells, and accumulation of immunosuppressive cells.^15,16^ The immune system can be exploited for therapy, as described in the Immunotherapy section.

Schwann cells are present in the NB-TME and are associated with favorable outcomes. They produce anti-angiogenic factors such as pigment epithelium-derived factors, tissue inhibitors of metalloproteinase-2 (TIMP-2), and secreted protein acidic and rich in cysteine (SPARC). SCs are inversely correlated with CAFs, suggesting a role in preventing the activation of fibroblasts.^75,76^ The latter derive from different cell types after exposure to transforming growth factor beta (TGF-β), interleukin (IL)-6, IL-8, tumor necrosis factor (TNF), or DNA damage. These pro-inflammatory factors interact with TAMs and contribute to escape from chemo- and immunotherapy.^74^ Mesenchymal stroma cells can promote cancer cells survival, growth, endothelial to mesenchymal (EMT) transition, and support hematopoiesis in bone marrow, the primary metastasis site in high-risk NB patients.^77^

Cancer cells coordinate the TME to face hypoxic conditions by promoting angiogenesis and regulating nutrient supply. In fact, hypoxia contributes to tumor progression and dedifferentiation, and decreases expression of neuronal and neuroendocrine markers, while inducing genes expressed in neural crest sympathetic progenitors.^78^

Besides the release of cytokines and other signaling molecules directly in the NB-TME, extracellular vesicles are also present in the tumors. These include exosomes, microvesicles, and large apoptotic bodies,^74^ containing integrins and other proteins involved in metabolic processes, and signaling pathways, including Epidermal growth factor (EGF,) IL-3, mammalian target of rapamycin (mTOR), and TNF-related apoptosis-inducing ligand (TRAIL). In fact, exosomes from *MYCN*-amplified cells have been reported to promote migration and chemoresistance of non-*MYCN*-amplified NB cells.^79^

## Precision Medicine for High-Risk Neuroblastoma

As mentioned above, the current treatment regimens have a severe impact on the normal development of surviving children. Thus, there is an urgent need to identify novel therapeutic targets and develop treatment strategies that are more effective and less toxic. In recent years, several small molecules that induce differentiation or disrupt metabolic pathways in NB have been identified. Moreover, immunotherapy has been postulated as a strong candidate with the latest progress in the field. A selection of preclinical NB models used to analyze these approaches including 2D cell cultures, 3D spheres/organoids, and in vivo models is presented in Supplementary Table S4.

### Targeting Metabolism

**Figure 3.**
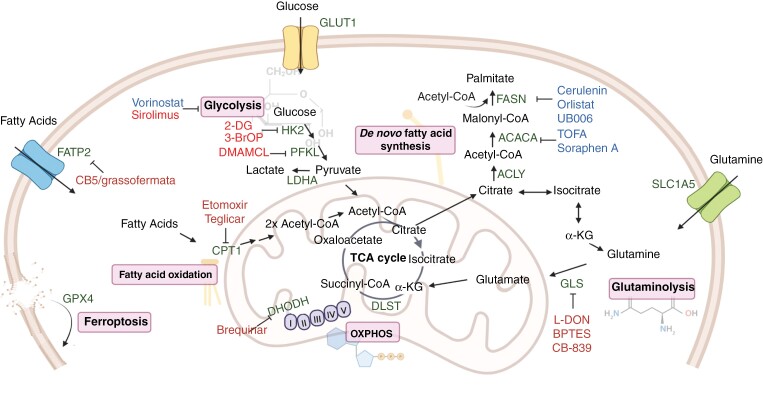
Metabolic targets and their inhibitors. Several enzymes participating in energy metabolic processes have been described to be upregulated in NB, conferring survival advantage. Inhibitors of these targets have been associated with reduced proliferation, and, in some cases, also in induction of differentiation. 2-DG: 2-deoxyglucose; 3-BrOP: 3-bromo-2-oxopropionate-1-propyl ester; HK2: hexokinase 2; PFKL: phosphofructokinase, liver type; LDHA: lactate dehydrogenase A; FATP2: fatty acid transport protein 2; GPX4: glutathione peroxidase 4; CPT1: carnitine/palmitoyl-transferase 1; DHODH: dihydroorotate dehydrogenase; DLST: dihydrolipoamide S-succinyltransferase; ACLY: ATP citrate lyase; ACACA: acetyl-CoA carboxylase A; FASN: fatty acid synthase; GLS: glutaminase; α-KG: α-ketoglutarate.

Cancer cells reprogram their metabolism to sustain cell proliferation and adapt to conditions of nutrient and oxygen deprivation. The cancer metabolism field has grown during the last decade, but only recently, alterations in the metabolism of NB cells and their implications in tumorigenesis have started to be defined. As MYCN is a master regulator of a vast number of metabolic enzymes, its amplification has profound effects on both anabolic and catabolic metabolic processes.^80^ The metabolic targets described in NB and their inhibitors are illustrated in Figure 3.

#### Glycolysis and oxidative phosphorylation (OXPHOS).

Glucose consumption is enhanced in NB tumors with *MYCN* amplification. Several of the enzymes participating in glucose metabolism are directly regulated by this oncoprotein, as well as by the hypoxia inducible factor 1-α (HIF1-α), resulting in increased glycolytic flux.^80,81^ For instance, the expression of the rate-limiting enzyme hexokinase 2 (HK2), the glucose transporter (GLUT1), and the final enzyme in the pathway, lactate dehydrogenase A (LDHA), producing lactate from pyruvate is higher in *MYCN*-amplified compared to non-*MYCN*-amplified NB.^82^ Several inhibitors targeting glycolysis at different levels either alone or in combination with other compounds, including 2-deoxyglucose (2-DG), dimethylaminomicheliolide (DMAMCL), and 3-BrOP (3-bromo-2-oxopropionate-1-propyl ester) have shown efficacy in both in vitro as well as in vivo NB models.^83–85^

Moreover, as MYCN increases expression of several enzymes in the tricarboxylic acid (TCA) cycle and in OXPHOS, several studies have demonstrated that inhibition of mitochondrial respiration is a vulnerability in high-risk NB. Depletion of dihydrolipoamide S-succinyltransferase (DLST), an enzyme in the α-ketoglutarate (α-KG) dehydrogenase complex, repressed nicotinamide adenine dinucleotide (NADH) production and disrupted OXPHOS, leading to apoptosis and arrest in NB cells.^86^ In addition, treatment with the histone deacetylase (HDAC) inhibitor vorinostat inhibited MYCN and glycolysis while increasing fatty acid oxidation (FAO) as well as OXPHOS as a compensatory mechanism for ATP production. The combination of vorinostat and mTOR inhibitor sirolimus, also downregulated glycolytic enzymes and resulted in increased FAO and OXPHOS, as well as elevated production of reactive oxygen species (ROS). Both these drugs reduced tumor burden in a xenograft model for NB.^87^

#### Amino acid and nucleotide metabolism.

Amplification of the *MYCN* oncogene results in the upregulation of several essential amino acid transporters, including the solute carriers SLC7A5 and SLC43A1, by directly binding to their promoters. However, if the levels of these transporters are reduced, MYCN is also downregulated, demonstrating a negative feedback-loop.^88^

Glutamine consumption is enhanced in cancer cells,^89^ and high expression of SLC1A5 (*ASCT2*), the major glutamine transporter, is correlated both to the levels of MYCN as well as the activating transcription factor 4 (ATF4) in NB cells. Glutamine deprivation or downregulation of SLC1A5 induced apoptosis in *MYCN*-amplified NB cells and prevented tumor formation in a xenograft model.^90,91^ Notably, the rate-limiting enzyme of glutaminolysis, glutaminase (GLS), converting glutamine into glutamate, is directly regulated by MYCN^92^ and, unexpectedly, we found that *MYCN-*amplified NB cells synthesized glutamine de novo thus contributing to aggressiveness of these cells.^80^ Glutamate can further be included in the TCA cycle to obtain energy. Overexpression of *MYCN* in non-*MYCN*-amplified NB cells sensitized treatment with the GLS inhibitor L-6-diazo-5-oxo-L-norleucine (L-DON).^93^ Thus, c-MYC as well as MYCN have been described to induce glutamine addiction in cancer cells, with enhanced glutaminolysis and a crucial role in cancer cell survival.

The serine/glycine/one-carbon (SGOC) metabolic pathway generates different macromolecules including nucleotides, lipids, and proteins, and is of importance for redox homeostasis and methylation. *MYCN*-amplified NB cells with activating transcription factor 4 (ATF4) expression showed increased transcriptional activation of this metabolic pathway and small molecules targeting SGOC-induced metabolic stress and autophagy in cell lines and xenograft models.^94^ Moreover, MYCN was shown to directly activate the expression of methylenetetrahydrofolate dehydrogenase 1 (MTHFD1), an important enzyme in the folate cycle, maintaining NADPH redox homeostasis in *MYCN*-amplified NB. Its knockdown increased the levels of ROS, induced apoptosis, and enhanced the antitumor effect of the bromodomain and extra-terminal (BET) inhibitor JQ1, indicating its potential as an oncogene to target in NB.^95^

Furthermore, increased levels of enzymes participating in the synthesis of purines and pyrimidines are correlated to *MYCN* amplification. One example is dihydroorotate dehydrogenase (DHODH),^96^ which has been proposed as an independent prognostic marker for NB.^97^ The use of DHODH inhibitors such as brequinar resulted in reduced NB tumorigenesis. Simultaneous treatment together with dipyridamole, an inhibitor of nucleoside transport, synergized to suppress NB growth and to overcome resistance to DHODH inhibition.^96^ Moreover, combining brequinar and the alkylating agent temozolomide showed curative effects in TH-*MYCN* mice. However, the use of brequinar as a single agent clinically has been limited both due to its narrow therapeutic window as well as toxicity. Still, combination of brequinar with other strategies could reduce adverse effects by allowing for lower doses of the individual agents while enhancing therapeutic efficacy. One other attractive possibility would be the development of more specific and less toxic DHODH inhibitors.

#### Lipid metabolism

We have identified fatty acids as the preferred fuel to obtain mitochondrial ATP in *MYCN*-amplified NB cells. We also showed that inhibition of carnitine/palmitoyl-transferase 1 (CPT1), the rate-limiting enzyme of FAO, reduced proliferation in vitro and tumor growth in vivo.^80^ Moreover, inhibition of MYCN impaired β-oxidation of fatty acids leading to the accumulation of lipid droplets.^98^ Tao et al., reported that *MYCN* amplification promoted fatty acid uptake and biosynthesis due to direct upregulation of the fatty acid transport protein 2 (FATP2). Downregulation of *SLC27A2,* encoding FATP2, reduced tumor burden, and exerted a synergistic effect in preventing tumor progression in different NB mouse models when combined with conventional chemotherapeutic drugs.^99^

Cancer cells have an aberrant activation of de novo fatty acid synthesis contributing to their survival by providing macromolecules important for biological membranes, ATP production, and lipid trafficking regulating many signaling pathways.^100^ Thus, inhibition of this metabolic process is a potential approach for targeting tumors. Notably, MYCN upregulates enzymes participating in lipogenesis including fatty acid synthase (FASN) and acetyl-CoA carboxylase A (ACACA). We have shown that inhibition of these enzymes induced differentiation in NB cell lines as well as in xenograft tumors and reduced tumor burden in mouse models in vivo.^101^

#### Challenges and opportunities of metabolic inhibitors

The metabolic reprogramming observed in NB has proven a vulnerability for treatment and development of new precision medicine strategies, many of which show promising results in preclinical settings. However, despite successful in these studies, translating them to clinical practice has faced several challenges. For instance, the high concentration of these enzymes in cancer cells requires high drug levels to achieve a response that could neutralize the target, which presents additional pharmacokinetic issues and possible off-target effects. Additionally, due to the ability of cancer cells to reprogram their metabolic pathways, targeting one specific enzyme could lead to development of resistance. In this regard, combination therapies show an advantage over single treatments,^102^ and further research will be key in determining their therapeutic potential.^103^ Moreover, while some of these compounds have antiproliferative effects, others such as drugs targeting lipid metabolism surprisingly demonstrated potential as differentiation-inducing agents.^101^*MYCN*-amplified NB cells exhibit enhanced metabolism driven by the upregulation of several enzymes, adaptations which are essential for supporting their rapid proliferation and survival. Although more mechanistic studies are required to understand the interplay between metabolism and differentiation, it is tempting to speculate that some metabolic pathways are crucial for maintaining the undifferentiated state. In the case of inhibitors targeting the lipid synthesis enzymes ACACA and FASN, disrupting lipid metabolism and signaling, as well as influencing membrane composition and dynamics, could have a strong impact in inducing a differentiated state of *MYCN*-amplified NB cells. In addition, inhibiting fatty acid synthesis alters the balance of acetyl coenzyme A (acetyl-CoA) usage, leading to reduced histone acetylation, which in turn has been associated with the induction of differentiation from neural progenitors into neurons.^104^

### Targeting the Antioxidant Systems

Metabolism is closely connected to the production of ROS, which in turn are controlled by the antioxidant systems. Several studies have focused on cysteine, the rate-limiting amino acid for synthesis of glutathione (GSH), an antioxidant important for the clearance of ROS. Upon cysteine deprivation, MYCN induced lipid peroxidation and increased the sensitivity towards ferroptosis. The glutathione peroxidase 4 (GPX4) enzyme protects against lipid peroxides, one type of ROS. Combined inhibition of cysteine uptake or transsulfuration, and downregulation of GPX4 resulted in tumor regression in a NB mouse model.^105^ Buthionine sulfoximine (BSO), a selective inhibitor of γ-glutamylcysteine synthetase (γ-GCS), the rate-limiting enzyme in GSH synthesis, has been used in a clinical trial in patients with recurrent NB in combination with melphalan with promising results.^106^

We found that metabolic reprogramming by MYCN inhibition in *MYCN*-amplified NB is accompanied by an increase in antioxidant enzymes.^80^ Additionally, a recent study showed that accumulation of 7-dehydrocholesterol (7-DHC), the substrate of 7-DHC reductase (DHCR7), prevented ferroptosis in NB cells, inducing a resistant phenotype associated with aggressiveness.^107^ Both mechanisms reveal possible vulnerabilities that could be exploited for therapy.

### Differentiation Therapies: Retinoic Acid and Potential Alternative Approaches

Differentiation therapy demonstrates significant potential as a strategy for cancer treatment, as it reactivates cellular pathways that will force cancer cells to mature into a nonaggressive differentiated phenotype. This is especially promising for 4S stage patients, in which tumors spontaneously regress via cell differentiation. Two main cell identities have been described in NB according to their differentiation state: the undifferentiated mesenchymal (MES) and the committed adrenergic (ADRN) phenotype.^108^ These differ in their mRNA signatures, and in the resistance to chemotherapeutic drugs, where ADRN cells are more sensitive than MES. As NB cells show plasticity, they have the capacity to undergo epigenetic reprogramming and transition from the MES to the ADRN state.^21^ Notably, such plasticity has recently also been identified in NB tumors, as they can change between identities via an intermediate “transitional” state.^17,19^ This switch can be triggered by therapy, as the enhancer of zeste homolog 2 inhibitor tazemetostat, approved for sarcoma treatment, which has been shown to reprogram cells from a MES to an ADRN state.^109^ On the other hand, retinoids rewire the enhancer landscape, establishing a retinosympathetic core regulatory circuitry characterized by decreased MYCN levels and proliferation, followed by induction of apoptosis and differentiation.^110^ Combination of retinoic acid (RA) and the CDK4/6 inhibitor palbociclib (the latter used for breast cancer), triggered differentiation of ADRN cell lines and reset their oncogenic regulatory circuit.^111^ It is possible that NB acquires more cellular states, and future studies will determine the role of plasticity in progression and treatment.

Strategies aimed at inducing differentiation are believed to be less damaging to normal cells than common chemotherapeutic agents, making them a preferable option for managing childhood cancers. However, differentiation agents sometimes fail and do not reach clinical practice, either due to high toxicity, resistance to therapy, lack of stratification of patients, or treatment timing. Differentiation therapies, specifically RA, are commonly used during the maintenance phase but might be more effective at early stages of cancer development. A recent study characterized the proteome and phospho-proteome of NB cell lines upon differentiation with all-*trans* retinoic acid (ATRA), identifying alterations in pathways related to cytoskeleton organization, cell division, chaperone function, protein folding, as well as one-carbon metabolism.^112^

Isotretinoin (13-*cis*-RA) is the isoform used clinically as it has the best pharmacokinetic profile, even if its association with the RA receptors is weaker.^113^ For instance, 9-*cis* RA is more potent than isotretinoin but shows higher toxicity, shorter half-life, and lower bioavailability, factors limiting its therapeutic potential.^114^ Several clinical trials have been performed with 13-*cis* RA, ATRA, or fenretinide, alone or in combination with other strategies.^115^ For instance, 13-*cis* RA increased event-free survival in high-risk NB patients who had undergone either autologous bone marrow transplantation or chemotherapy.^116^ The main issue of these compounds is that they are pan-activators of RA signaling and can result in several side effects. Notably, patients were not stratified according to RAR expression levels, and thus an accurate prediction of their response to treatment was compromised.^117,118^ This might explain the questioned efficacy and the fact that half of the patients treated with 13-*cis*-RA still relapsed.^119^ Hence, analysis of biomarkers and patient stratification, together with combination therapies and better delivery systems, may improve the efficacy of RA for NB treatment (reviewed in the study by Giuli et al.^120^).

In addition, several other strategies have been tested with the aim to induce differentiation in NB cells, but more efforts are needed to bring them to clinical trials as viable alternatives to RA. MYCN is a key player in maintaining an undifferentiated phenotype in NB and its genetic targeting or inhibition drives differentiation. MYCN downregulation precedes RA-induced differentiation^121^ and its genetic silencing via siRNA or inhibition with the BET inhibitor JQ1 resulted in neuronal differentiation in vitro and reduced tumor burden in vivo.^122,123^ The newer IBET-762 and OTX-015 BET inhibitors were tested in clinical trials for the treatment of hematopoietic and solid tumors. However, due to adverse effects, they were discontinued, indicating high toxicity.^124^ The MYC dominant negative Omomyc peptide caused reduced growth and apoptosis in SH-SY5Y NB cells, which do not express MYCN but high c-MYC levels. Notably, a lower impact was shown for SHEP cells that similar to SH-SY5Y do not express MYCN but in contrast have normal c-MYC levels. These results suggest that Omomyc function is dependent on MYC expression levels.^125,126^ Future research will reveal whether targeting MYCN with either Omomyc or the third-generation BET inhibitors (eg, ABBV-075 or TEN-010), alone or in combination with other strategies has potential for clinical practice. We have shown that MYCN can also be targeted by disrupting the binding to its partner MAX by the small molecule inhibitors 10058-F4 or 10074-G5, resulting in neuronal differentiation of *MYCN*-amplified NB cells in vitro as well as increased survival of TH-*MYCN* mice.^98,127^ In addition, the MYCN-specific anti-gene peptide nucleic acid (agPNA) oligonucleotide BG002 in combination with RA led to differentiation of *MYCN*-amplified cell lines.^128^ Moreover, our work showed that MYCN regulates the *miR-17~92* microRNA cluster, which subsequently represses several target genes including the estrogen receptor α (ERα) and the glucocorticoid receptor (GR). Activation of these receptors with 17-β-estradiol, the ligand for ERα, and with dexamethasone, the synthetic ligand for GR, alone or in combination, resulted in NB differentiation, especially when combined with all-*trans* RA.^118,129–131^

Histone deacetylase inhibitors (HDACi) including valpronic acid (VPA), vorinostat (SAHA), or sodium phenylbutyrate (4-PB), are agents described to induce NB differentiation. These compounds are in clinical trials in combination with immunotherapy, radiation, or other agents: isotretinoin, the proteasome inhibitor bortezomib, the mTOR inhibitor temsirolimus,^132^ or the checkpoint inhibitor nivolumab.^133^ Notably, RA triggered neural differentiation in NB by promoting H3K14 acetylation and mitochondrial function.^134^

Niclosamide ethanolamine (NEN) can drive NB differentiation by affecting chromatin structure. This compound increased mitochondrial respiration, causing upregulation of the NAD^+^/NADH and α-KG/2-hydroxyglutarate (2-HG) ratio, which in turn resulted in promoter CpG island demethylation, activating the differentiation program in *MYCN*-amplified NB.^135^ In addition, we previously showed that inhibition of fatty acid synthase (FASN) or acetyl-CoA carboxylase A (ACACA) with TOFA, Cerulenin, Orlistat (approved for treatment of obesity), Soraphen A, or UB006 (Figure 3), induced NB differentiation independently of *MYCN* status.^101^

Moreover, inhibition of the Rho guanine nucleotide exchange factor 12 (ARHGEF12) by the pharmacological inhibitor Y16 or by shRNA robustly promoted NB differentiation and reduced tumorigeneicity through Ras homolog familiy member A(RhoA)/Rho associated protein kinase (ROCK) signaling.^136^ Furthermore, the small imipridone molecule ONC201 originally identified as a TRAIL-activating compound, which impairs mitochondrial respiration^137,138^ resulted in neurite outgrowth in *MYCN*-amplified cell lines. Interestingly, treatment with ONC201 and 2-DG induced combined metabolic rewiring, leading to a synergistic anticancer effect.^137^ ONC201 is both an antagonist of the dopamine receptor D2 (DRD2) as well as an allosteric agonist of the mitochondrial protease caseinolytic mitochondrial matrix peptidase proteolytic subunit (ClpP). This compound is currently in phase I and II clinical trials for diffuse intrinsic pontine glioma (DIPG) and recurrent/refractory H3K27M glioma, glioblastoma, acute leukemia, multiple myeloma, as well as breast, colorectal, and neuroendocrine tumors.^138,139^

### Immunotherapy

The multimodal approach used for high-risk NB patients includes immunotherapy as part of the maintenance phase for any minimal residual disease together with RA. It has become a revolutionary strategy to target several adult tumors as well as childhood hematological malignancies. However, immune checkpoint inhibitors have not been as successful in solid childhood tumors in part due to their low mutational profile.^3^

The disialoganglioside GD2 is highly expressed in NB, while only elevated in normal cells from neuroectodermal origin including neurons, skin melanocytes, and pain fibers, making it a potential therapeutic target.^140^ In fact, the use of monoclonal antibodies (mAbs) against GD2 increased the event-free survival rate of patients by 20%. Dinutuximab (ch14.18) was the first GD2 mAb approved by the Food and Drug Administration (FDA) as well as for treatment of pediatric cancer. In early protocols it was combined with granulocyte-macrophage colony stimulating factor (GM-CSF), IL-2, and 13-*cis*-RA for treatment of high-risk NB responding to the multimodal treatment.^141^ However, the combination with IL-2 showed significant toxicity associated with dosing challenges which led to adverse events and mortality in some cases. Later studies demonstrated that there was no clear benefit of IL-2 addition to the regimen, and thus, it was omitted and is not included in current protocols.^69^ A second anti-GD2 mAb, naxitamab, which combined with GM-CSF, is used for the treatment of high-risk NB patients with relapsed or refractory disease limited to the bone or bone marrow.^142^

Yet, there are still some issues to consider regarding the use of immunotherapy in NB patients. Administration of anti-GD2 mAbs has adverse effects including pain, hypersensitivity, fever, neurotoxicity, allergic reactions, and gastrointestinal symptoms. Due to the severe dose-dependent pain, multiagent analgesics are infused at the same time for alleviation.^143^ In addition, even though anti-GD2 mAbs have increased the survival rate of patients, the prognosis is still near 50% survival in high-risk NB, with a high relapse rate. A phase I clinical trial using the GD2/GD3 vaccine in NB patients with a history of disease progression showed safety and increased survival. The efficacy of ganglioside vaccines to stimulate a robust immune response was explored in a phase II clinical trial, which indicated that the vaccine was well-tolerated and led to a notable antibody response, contributing to prolonged survival in some of the patients.^144^ Recently, it was demonstrated that the use of oral β-glucan as an adjuvant improved the efficacy of ganglioside vaccines by increasing the IgG antibody response without additional toxic effects.^145^

The immune checkpoint molecule B7-H3 CD276 is highly expressed in NB and in other pediatric solid tumors, compared to normal tissues. The B7-H3-targeting MGA271 antibody (enoblituzumab), approved for prostate cancer with biochemical recurrence, has also been clinically analyzed in children with NB (NCT02982941), however, no results have as of yet been reported. A clinical study using compartmental radioimmunotherapy (cRIT) with the anti-B7H3 murine monoclonal antibody omburtamab showed both safety and therapeutic efficacy in metastatic NB.^146^ Bispecific antibodies are being developed, including anti-GD2 anti-B7-H3 antibodies. By targeting two tumor antigens, these antibodies are more specific and can decrease the binding to peripheral nerves thus reducing pain.^147^ Nevertheless, finding novel immunotherapeutic approaches in NB remains challenging for several reasons. As mentioned earlier, childhood solid tumors have a low mutational burden which in turn results in weak T-cell infiltration. However, recent bulk- and scRNA-sequencing analysis revealed T-cell infiltration in tumors that increased after exposure to chemotherapy.^148^ The lower expression of MHC-I in NB, along with various immune evasion strategies, may account for the low infiltration and responsiveness of immune cells.^149^ Hence, understanding the immunogenic profile of NB will be key for achieving clinical success through the development of different targeted immunotherapeutic strategies.

None of the clinical trials including T-cells engineered chimeric antigen receptors (CAR-T) have shown promising results in NB due to a reduction in the number of cells after administration and the evading nature of the immune system.^150^ However, the more sophisticated designs of next-generation CAR-T-cells targeting GD2 are under development.^151,152^ As an example, GD2-targeting CAR-T-cells (GD2-CART01) recently shown to be safe and associated with sustained tumor eradication in several cases in a phase I/II clinical trial for relapsed and refractory high-risk NB.^153^ In addition, B7-H3 CAR-T cells showed promising results in preclinical studies and are currently investigated in a phase I clinical trial (NCT04483778). The cell surface proteoglycan Glypican-2 (GPC2) is particularly overexpressed in *MYCN*-amplified NB and has emerged as a novel target for CAR-T cell therapy.^154,155^ Recently, it was reported that peptide-centric CARs (PC-CARs) against PHOX2B selectively eliminated NB tumors. This innovative approach paves the way for targeting of non-immunogenic intracellular oncoproteins displayed by MHC molecules on the tumor cell surface, potentially expanding the range of targetable antigens.^156^ Moreover, CAR-T cells targeting both GD2 and the immune checkpoint inhibitor B7H3 (CD276) have shown antitumoral effects in mouse models, with improved metabolic function and reduced risk of neurotoxicity.^157^ Combination treatment of dinutuximab with chemotherapeutic drugs is also under investigation. Administration of irinotecan, temozolomide, and dinutuximab with GM-CSF in patients with relapsed or refractory NB showed significant treatment response.^158^ Even though the results of immune checkpoint inhibitors (ICI) monotherapy were disappointing, such as the anti-PD-1 inhibitor nivolumab,^159^ a combination of several ICIs could enhance T cell immunity and the focus for ongoing clinical trials.^160,161^ In addition, a recent study showed that NB with a MES lineage signature exhibited higher immunogenicity than those with an ADRN profile.^162^ Mesenchymal linage cells promoted T cell infiltration and were sensitive to cytotoxic T and NK cells, and to anti-PD-1 as well as anti-CTL4 antibodies, thus highlighting the potential for more effective immunotherapy strategies tailored to specific cellular states within the tumor. Activation of NK cells induced death in NB cells in vitro, however, similar as for T cells, a low infiltration of NK cells was reported in tumors.^163^ In addition, increased MYCN levels using an inducible in vitro system led to downregulation of NK-activating ligands.^164^ In patients, immune escape mechanisms are responsible for impaired NK cell function. Administration of ex vivo expanded NK cells together with GD2 mAbs has been investigated in NB cells although more studies are needed to evaluate this approach.^165^ A recent study using scRNA sequencing of 24 NB tumors prior to and after chemotherapy described the interaction between the immune components of these tumors. The authors identified NECTIN2-TIGIT (Nectin-2-immune checkpoint protein/T cell immunoglobin and ITM domain) as an important immune checkpoint and showed that combining TIGIT and PD-L1 blockade reduced NB growth and induced complete responses in preclinical models. These findings provide promising insights for immunotherapy combination treatments.^166^

The “cold” immunogenic environment of NB tumors makes the application of immune-based therapies challenging. Exposure to several strategies comprising of multiple immunological mechanisms that can stimulate the immune system could however result in a more effective outcome (Figure 2).

## Conclusions and Future Perspectives

Neuroblastoma is a highly heterogeneous disease, and in the most aggressive high-risk cases, the current treatment options are insufficient. Thus, understanding the biology causing these tumors is needed for the development of new approaches. With the combination of novel wet-lab techniques and bioinformatic tools, new insights into the landscape of NB, concerning the cellular origin and the microenvironment have been gained. In addition, the generation of experimental models that mimic the human disease has improved knowledge, from tumor formation and progression to treatment resistance. Identification of high levels of GD2 in NB was key for the development of the first immunotherapy with successful clinical outcomes. Moreover, induction of differentiation has long been used for the maintenance phase in high-risk NB patients. Notably, recent studies have focused on deciphering the behavior of the most aggressive cases of this childhood tumor, including the interaction with the NB-TME as well as inhibition of differentiation and reprogramming of cancer metabolism, providing a basis for novel combinatory therapies. Targeting different metabolic enzymes has been demonstrated to enhance neural differentiation. Overall, the complexity of NB calls for a combination of multimodal and personalized therapies to improve patient survival and reduce therapy resistance. The future holds great promise, as significant research efforts are directed towards bringing these therapeutic approaches to clinical practice.

## Supplementary material

Supplementary material is available online at *Neuro-Oncology* (https://academic.oup.com/neuro-oncology).

noae152_suppl_Supplementary_Material

## Data Availability

No new data were generated or analyzed in support of this review article.

## References

[1] Siegel DA , King JB, Lupo PJ, et al Counts, incidence rates, and trends of pediatric cancer in the United States, 2003-2019. J Natl Cancer Inst. 2023;115(11):1337–1354. 37433078 10.1093/jnci/djad115 PMC11018256

[2] Steliarova-Foucher E , Colombet M, Ries LAG, et al; IICC-3 contributors. International incidence of childhood cancer, 2001-10: a population-based registry study. Lancet Oncol. 2017;18(6):719–731. 28410997 10.1016/S1470-2045(17)30186-9 PMC5461370

[3] Gröbner SN , Worst BC, Weischenfeldt J, et al; ICGC PedBrain-Seq Project. The landscape of genomic alterations across childhood cancers. Nature. 2018;555(7696):321–327. 29489754 10.1038/nature25480

[4] Brodeur GM , Seeger RC, Barrett A, et al International criteria for diagnosis, staging, and response to treatment in patients with neuroblastoma. J Clin Oncol. 1988;6(12):1874–1881. 3199170 10.1200/JCO.1988.6.12.1874

[5] Brodeur GM. Spontaneous regression of neuroblastoma. Cell Tissue Res. 2018;372(2):277–286. 29305654 10.1007/s00441-017-2761-2 PMC5920563

[6] Maris JM , Hogarty MD, Bagatell R, Cohn SL. Neuroblastoma. Lancet. 2007;369(9579):2106–2120. 17586306 10.1016/S0140-6736(07)60983-0

[7] Smith V , Foster J. High-risk neuroblastoma treatment review. Children (Basel). 2018;5(9):114. 30154341 10.3390/children5090114 PMC6162495

[8] Friedman DN , Henderson TO. Late effects and survivorship issues in patients with neuroblastoma. Children (Basel). 2018;5(8):107. 30082653 10.3390/children5080107 PMC6111874

[9] Rothstein M , Simoes-Costa M. On the evolutionary origins and regionalization of the neural crest. Semin Cell Dev Biol. 2023;138:28–35. 35787974 10.1016/j.semcdb.2022.06.008

[10] Kildisiute G , Kholosy WM, Young MD, et al Tumor to normal single-cell mRNA comparisons reveal a pan-neuroblastoma cancer cell. Sci Adv. 2021;7(6):eabd3311. 33547074 10.1126/sciadv.abd3311 PMC7864567

[11] Bedoya-Reina OC , Li W, Arceo M, et al Single-nuclei transcriptomes from human adrenal gland reveal distinct cellular identities of low and high-risk neuroblastoma tumors. Nat Commun. 2021;12(1):5309. 34493726 10.1038/s41467-021-24870-7 PMC8423786

[12] Jansky S , Sharma AK, Körber V, et al Single-cell transcriptomic analyses provide insights into the developmental origins of neuroblastoma. Nat Genet. 2021;53(5):683–693. 33767450 10.1038/s41588-021-00806-1

[13] Dong R , Yang R, Zhan Y, et al Single-cell characterization of malignant phenotypes and developmental trajectories of adrenal neuroblastoma. Cancer Cell. 2020;38(5):716–733.e6. 32946775 10.1016/j.ccell.2020.08.014

[14] Kameneva P , Artemov AV, Kastriti ME, et al Single-cell transcriptomics of human embryos identifies multiple sympathoblast lineages with potential implications for neuroblastoma origin. Nat Genet. 2021;53(5):694–706. 33833454 10.1038/s41588-021-00818-x PMC7610777

[15] Verhoeven BM , Mei S, Olsen TK, et al The immune cell atlas of human neuroblastoma. Cell Rep Med. 2022;3(6):100657. 35688160 10.1016/j.xcrm.2022.100657 PMC9245004

[16] Costa A , Thirant C, Kramdi A, et al Single-cell transcriptomics reveals shared immunosuppressive landscapes of mouse and human neuroblastoma. J ImmunoTher Cancer. 2022;10(8):e004807. 36054452 10.1136/jitc-2022-004807 PMC9362821

[17] Yuan X , Seneviratne JA, Du S, et al Single-cell profiling of peripheral neuroblastic tumors identifies an aggressive transitional state that bridges an adrenergic-mesenchymal trajectory. Cell Rep. 2022;41(1):111455. 36198269 10.1016/j.celrep.2022.111455

[18] Liu Q , Wang Z, Jiang Y, et al Single-cell landscape analysis reveals distinct regression trajectories and novel prognostic biomarkers in primary neuroblastoma. Genes & Dis 2022;9(6):1624–1638. 10.1016/j.gendis.2021.12.020 PMC9485279 36157484

[19] Thirant C , Peltier A, Durand S, et al Reversible transitions between noradrenergic and mesenchymal tumor identities define cell plasticity in neuroblastoma. Nat Commun. 2023;14(1):2575. 37142597 10.1038/s41467-023-38239-5 PMC10160107

[20] Furlan A , Dyachuk V, Kastriti ME, et al Multipotent peripheral glial cells generate neuroendocrine cells of the adrenal medulla. Science. 2017;357(6346):eaal3753. 28684471 10.1126/science.aal3753 PMC6013038

[21] van Groningen T , Koster J, Valentijn LJ, et al Neuroblastoma is composed of two super-enhancer-associated differentiation states. Nat Genet. 2017;49(8):1261–1266. 28650485 10.1038/ng.3899

[22] Körber V , Stainczyk SA, Kurilov R, et al Neuroblastoma arises in early fetal development and its evolutionary duration predicts outcome. Nat Genet. 2023;55(4):619–630. 36973454 10.1038/s41588-023-01332-y PMC10101850

[23] Gundem G , Levine MF, Roberts SS, et al Clonal evolution during metastatic spread in high-risk neuroblastoma. Nat Genet. 2023;55(6):1022–1033. 37169874 10.1038/s41588-023-01395-x PMC11481711

[24] Brodeur GM , Pritchard J, Berthold F, et al Revisions of the international criteria for neuroblastoma diagnosis, staging, and response to treatment. J Clin Oncol. 1993;11(8):1466–1477. 8336186 10.1200/JCO.1993.11.8.1466

[25] Maris JM , Weiss MJ, Guo C, et al Loss of heterozygosity at 1p36 independently predicts for disease progression but not decreased overall survival probability in neuroblastoma patients: a children’s cancer group study. J Clin Oncol. 2000;18(9):1888–1899. 10784629 10.1200/JCO.2000.18.9.1888

[26] Bown N , Cotterill S, Lastowska M, et al Gain of chromosome arm 17q and adverse outcome in patients with neuroblastoma. N Engl J Med. 1999;340(25):1954–1961. 10379019 10.1056/NEJM199906243402504

[27] Komotar RJ , Otten ML, Starke RM, Anderson RCE. Chromosome 1p and 11q deletions and outcome in neuroblastoma—a critical review. Clin Med Oncol. 2008;2(January–December):419–420. 21892309 10.4137/cmo.s391 PMC3161664

[28] Shimada H , Ambros IM, Dehner LP, et al The international neuroblastoma pathology classification (the Shimada system). Cancer. 1999;86(2):364–372. 10421273

[29] Cohn SL , Pearson ADJ, London WB, et al; INRG Task Force. The International Neuroblastoma Risk Group (INRG) classification system: an INRG task force report. J Clin Oncol. 2009;27(2):289–297. 19047291 10.1200/JCO.2008.16.6785 PMC2650388

[30] Shimada H , Ikegaki N. Genetic and histopathological heterogeneity of neuroblastoma and precision therapeutic approaches for extremely unfavorable histology subgroups. Biomolecules. 2022; 12(1):79. 35053227 10.3390/biom12010079 PMC8773700

[31] National Cancer Institute. Neuroblastoma Treatment (PDQ®)–Health Professional Version. Accessed June 30, 2024. https://www.cancer.gov/types/neuroblastoma/hp/neuroblastoma-treatment-pdq 26389190

[32] Nuchtern JG , London WB, Barnewolt CE, et al A prospective study of expectant observation as primary therapy for neuroblastoma in young infants: a children’s oncology group study. Ann Surg. 2012;256(4):573–580. 22964741 10.1097/SLA.0b013e31826cbbbd PMC5665168

[33] Nickerson HJ , Matthay KK, Seeger RC, et al Favorable biology and outcome of stage IV-S neuroblastoma with supportive care or minimal therapy: a children’s cancer group study. J Clin Oncol. 2000;18(3):477–486. 10653863 10.1200/JCO.2000.18.3.477

[34] Friedman DL , Kadan-Lottick NS, Whitton J, et al Increased risk of cancer among siblings of long-term childhood cancer survivors: a report from the childhood cancer survivor study. Cancer Epidemiol Biomarkers Prev. 2005;14(8):1922–1927. 16103438 10.1158/1055-9965.EPI-05-0066

[35] Bosse KR , Maris JM. Advances in the translational genomics of neuroblastoma: from improving risk stratification and revealing novel biology to identifying actionable genomic alterations. Cancer. 2016;122(1):20–33. 26539795 10.1002/cncr.29706 PMC4707066

[36] Valentijn LJ , Koster J, Zwijnenburg DA, et al TERT rearrangements are frequent in neuroblastoma and identify aggressive tumors. Nat Genet. 2015;47(12):1411–1414. 26523776 10.1038/ng.3438

[37] Huang M , Weiss WA. Neuroblastoma and MYCN. Cold Spring Harb Perspect Med. 2013;3(10):a014415. 24086065 10.1101/cshperspect.a014415 PMC3784814

[38] Pugh TJ , Morozova O, Attiyeh EF, et al The genetic landscape of high-risk neuroblastoma. Nat Genet. 2013;45(3):279–284. 23334666 10.1038/ng.2529 PMC3682833

[39] Grandori C , Cowley SM, James LP, Eisenman RN. The Myc/Max/Mad network and the transcriptional control of cell behavior. Annu Rev Cell Dev Biol. 2000;16:653–699. 11031250 10.1146/annurev.cellbio.16.1.653

[40] Otte J , Dyberg C, Pepich A, Johnsen JI. MYCN function in neuroblastoma development. Front Oncol. 2020;10(624079):1–12. 33585251 10.3389/fonc.2020.624079 PMC7873735

[41] Zimmerman MW , Liu Y, He S, et al MYC drives a subset of high-risk pediatric neuroblastomas and is activated through mechanisms including enhancer hijacking and focal enhancer amplification. Cancer Discov. 2018;8(3):320–335. 29284669 10.1158/2159-8290.CD-17-0993 PMC5856009

[42] Suenaga Y , Nakatani K, Nakagawara A. De novo evolved gene product NCYM in the pathogenesis and clinical outcome of human neuroblastomas and other cancers. Jpn J Clin Oncol. 2020;50(8):839–846. 32577751 10.1093/jjco/hyaa097

[43] Liu Z , Chen SS, Clarke S, Veschi V, Thiele CJ. Targeting MYCN in pediatric and adult cancers. Front Oncol. 2020;10(623679):1–15. 33628735 10.3389/fonc.2020.623679 PMC7898977

[44] Garralda E , Beaulieu ME, Moreno V, et al MYC targeting by OMO-103 in solid tumors: a phase 1 trial. Nat Med. 2024;30(3):762–771. 38321218 10.1038/s41591-024-02805-1 PMC10957469

[45] Gonzalez Malagon SG , Lopez Muñoz AM, Doro D, et al Glycogen synthase kinase 3 controls migration of the neural crest lineage in mouse and Xenopus. Nat Commun. 2018;9(1):1126. 29555900 10.1038/s41467-018-03512-5 PMC5859133

[46] Cazes A , Lopez-Delisle L, Tsarovina K, et al Activated Alk triggers prolonged neurogenesis and Ret upregulation providing a therapeutic target in ALK-mutated neuroblastoma. Oncotarget. 2014;5(9):2688–2702. 24811913 10.18632/oncotarget.1883 PMC4058037

[47] Mossé YP , Laudenslager M, Longo L, et al Identification of ALK as a major familial neuroblastoma predisposition gene. Nature. 2008;455(7215):930–935. 18724359 10.1038/nature07261 PMC2672043

[48] Zhang S , Anjum R, Squillace R, et al The potent ALK inhibitor Brigatinib (AP26113) overcomes mechanisms of resistance to first- and second-generation ALK inhibitors in preclinical models. Clin Cancer Res. 2016;22(22):5527–5538. 27780853 10.1158/1078-0432.CCR-16-0569

[49] Goldsmith KC , Park JR, Kayser K, et al Lorlatinib with or without chemotherapy in ALK-driven refractory/relapsed neuroblastoma: phase 1 trial results. Nat Med. 2023;29(5):1092–1102. 37012551 10.1038/s41591-023-02297-5 PMC10202811

[50] Rosswog C , Fassunke J, Ernst A, et al Genomic ALK alterations in primary and relapsed neuroblastoma. Br J Cancer. 2023;128(8):1559–1571. 36807339 10.1038/s41416-023-02208-y PMC10070426

[51] Yue Z , Gao C, Xing T, et al Combined analysis of PHOX2B at two time points and its value for further risk stratification in high-risk neuroblastoma. Pediatr Blood Cancer. 2023;70(5):e30261. 36815592 10.1002/pbc.30261

[52] Pattyn A , Morin X, Cremer H, Goridis C, Brunet JF. The homeobox gene Phox2b is essential for the development of autonomic neural crest derivatives. Nature. 1999;399(6734):366–370. 10360575 10.1038/20700

[53] Cheung NKV , Zhang J, Lu C, et al Association of age at diagnosis and genetic mutations in patients with neuroblastoma. JAMA. 2012;307(10):1062–1071. 22416102 10.1001/jama.2012.228 PMC3527076

[54] George SL , Lorenzi F, King D, et al Therapeutic vulnerabilities in the DNA damage response for the treatment of *ATRX* mutant neuroblastoma. EBioMedicine. 2020;59(102971):1–12. 10.1016/j.ebiom.2020.102971 PMC7452577 32846370

[55] Zeineldin M , Federico S, Chen X, et al MYCN amplification and ATRX mutations are incompatible in neuroblastoma. Nat Commun. 2020;11(1):913. 32060267 10.1038/s41467-020-14682-6 PMC7021759

[56] Hartlieb SA , Sieverling L, Nadler-Holly M, et al Alternative lengthening of telomeres in childhood neuroblastoma from genome to proteome. Nat Commun. 2021;12(1):1269. 33627664 10.1038/s41467-021-21247-8 PMC7904810

[57] He J , Gu L, Zhang H, Zhou M. Crosstalk between MYCN and MDM2-p53 signal pathways regulates tumor cell growth and apoptosis in neuroblastoma. Cell Cycle. 2011;10(17):2994–3002. 21862876 10.4161/cc.10.17.17118 PMC3218600

[58] Ackermann S , Cartolano M, Hero B, et al A mechanistic classification of clinical phenotypes in neuroblastoma. Science. 2018;362(6419):1165–1170. 30523111 10.1126/science.aat6768 PMC7875194

[59] Tsai JW , Cejas P, Wang DK, et al FOXR2 is an epigenetically regulated pan-cancer oncogene that activates ETS transcriptional circuits. Cancer Res. 2022;82(17):2980–3001. 35802025 10.1158/0008-5472.CAN-22-0671 PMC9437574

[60] Schmitt-Hoffner F , van Rijn S, Toprak UH, et al FOXR2 stabilizes MYCN protein and identifies non-MYCN-amplified neuroblastoma patients with unfavorable outcome. J Clin Oncol. 2021;39(29):3217–3228. 34110923 10.1200/JCO.20.02540 PMC8500564

[61] Zeineldin M , Patel AG, Dyer MA. Neuroblastoma: when differentiation goes awry. Neuron. 2022;110(18):2916–2928. 35985323 10.1016/j.neuron.2022.07.012 PMC9509448

[62] Nuchtern JG , London WB, Barnewolt CE, et al A prospective study of expectant observation as primary therapy for neuroblastoma in young infants: a Children’s Oncology Group study. Ann Surg. 2012;256(4):573–580. 22964741 10.1097/SLA.0b013e31826cbbbd PMC5665168

[63] Holmes K , Pötschger U, Pearson ADJ, et al; International Society of Paediatric Oncology Europe Neuroblastoma Group (SIOPEN). Influence of surgical excision on the survival of patients with stage 4 high-risk neuroblastoma: a report from the HR-NBL1/SIOPEN study. J Clin Oncol. 2020;38(25):2902–2915. 32639845 10.1200/JCO.19.03117

[64] Granger MM , Naranjo A, Bagatell R, et al Myeloablative busulfan/melphalan consolidation following induction chemotherapy for patients with newly diagnosed high-risk neuroblastoma: children’s oncology group trial ANBL12P1. Transplant Cell Ther. 2021;27(6):490.e1–490.e8. 10.1016/j.jtct.2021.03.006 PMC8855886 33823167

[65] Garaventa A , Poetschger U, Valteau-Couanet D, et al Randomized trial of two induction therapy regimens for high-risk neuroblastoma: HR-NBL1.5 International Society of Pediatric Oncology European Neuroblastoma Group Study. J Clin Oncol. 2021;39(23):2552–2563. 34152804 10.1200/JCO.20.03144

[66] Ladenstein R , Valteau-Couanet D, Brock P, et al Randomized trial of prophylactic granulocyte colony-stimulating factor during rapid COJEC induction in pediatric patients with high-risk neuroblastoma: the European HR-NBL1/SIOPEN study. J Clin Oncol. 2010;28(21):3516–3524. 20567002 10.1200/JCO.2009.27.3524

[67] Park JR , Kreissman SG, London WB, et al Effect of tandem autologous stem cell transplant vs single transplant on event-free survival in patients with high-risk neuroblastoma: a randomized clinical trial. JAMA. 2019;322(8):746–755. 31454045 10.1001/jama.2019.11642 PMC6714031

[68] Chung C , Boterberg T, Lucas J, et al Neuroblastoma. Pediatr Blood Cancer. 2021;68(Suppl 2):e28473. 33818884 10.1002/pbc.28473 PMC8785544

[69] Ladenstein R , Pötschger U, Valteau-Couanet D, et al Interleukin 2 with anti-GD2 antibody ch14.18/CHO (dinutuximab beta) in patients with high-risk neuroblastoma (HR-NBL1/SIOPEN): a multicentre, randomised, phase 3 trial. Lancet Oncol. 2018;19(12):1617–1629. 30442501 10.1016/S1470-2045(18)30578-3

[70] Twist CJ , Schmidt ML, Naranjo A, et al Maintaining outstanding outcomes using response- and biology-based therapy for intermediate-risk neuroblastoma: a report from the children’s oncology group study ANBL0531. J Clin Oncol. 2019;37(34):3243–3255. 31386611 10.1200/JCO.19.00919 PMC6881103

[71] Borriello L , Seeger RC, Asgharzadeh S, DeClerck YA. More than the genes, the tumor microenvironment in neuroblastoma. Cancer Lett. 2016;380(1):304–314. 26597947 10.1016/j.canlet.2015.11.017 PMC5558454

[72] Tzinia AK , Kitsiou PV, Talamagas AA, Georgopoulos A, Tsilibary EC. Effects of collagen IV on neuroblastoma cell matrix-related functions. Exp Cell Res. 2002;274(2):169–177. 11900477 10.1006/excr.2001.5463

[73] Tadeo I , Berbegall AP, Castel V, et al Extracellular matrix composition defines an ultra-high-risk group of neuroblastoma within the high-risk patient cohort. Br J Cancer. 2016;115(4):480–489. 27415013 10.1038/bjc.2016.210 PMC4985353

[74] Blavier L , Yang RM, DeClerck YA. The tumor microenvironment in neuroblastoma: new players, new mechanisms of interaction and new perspectives. Cancers (Basel). 2020;12(10):2912. 33050533 10.3390/cancers12102912 PMC7599920

[75] Chlenski A , Liu S, Crawford SE, et al SPARC is a key schwannian-derived inhibitor controlling neuroblastoma tumor angiogenesis. Cancer Res. 2002;62(24):7357–7363. 12499280

[76] Huang D , Rutkowski JL, Brodeur GM, et al Schwann cell-conditioned medium inhibits angiogenesis in vitro and in vivo. Med Pediatr Oncol. 2000;35(6):590–592. 11107124 10.1002/1096-911x(20001201)35:6<590::aid-mpo21>3.0.co;2-o

[77] Hochheuser C , Windt LJ, Kunze NY, et al Mesenchymal stromal cells in neuroblastoma: exploring crosstalk and therapeutic implications. Stem Cells Dev. 2021;30(2):59–78. 33287630 10.1089/scd.2020.0142 PMC7826431

[78] Jögi A , Øra I, Nilsson H, et al Hypoxia alters gene expression in human neuroblastoma cells toward an immature and neural crest-like phenotype. Proc Natl Acad Sci U S A. 2002;99(10):7021–7026. 12011461 10.1073/pnas.102660199 PMC124521

[79] Fonseka P , Liem M, Ozcitti C, et al Exosomes from N-Myc amplified neuroblastoma cells induce migration and confer chemoresistance to non-N-Myc amplified cells: implications of intra-tumour heterogeneity. J Extracell Vesicles. 2019;8(1):1597614. 31007876 10.1080/20013078.2019.1597614 PMC6461098

[80] Oliynyk G , Ruiz-Pérez MV, Sainero-Alcolado L, et al MYCN-enhanced oxidative and glycolytic metabolism reveals vulnerabilities for targeting neuroblastoma. iScience. 2019;21(November 22):188–204. 31670074 10.1016/j.isci.2019.10.020 PMC6889365

[81] Qing G , Skuli N, Mayes PA, et al Combinatorial regulation of neuroblastoma tumor progression by N-Myc and hypoxia inducible factor HIF-1alpha. Cancer Res. 2010;70(24):10351–10361. 20961996 10.1158/0008-5472.CAN-10-0740 PMC3005134

[82] Li H , Yang F, Hu A, et al Therapeutic targeting of circ-CUX1/EWSR1/MAZ axis inhibits glycolysis and neuroblastoma progression. EMBO Mol Med. 2019;11(12):e10835. 31709724 10.15252/emmm.201910835 PMC6895612

[83] Hagenbuchner J , Kiechl-Kohlendorfer U, Obexer P, Ausserlechner MJ. BIRC5/Survivin as a target for glycolysis inhibition in high-stage neuroblastoma. Oncogene. 2016;35(16):2052–2061. 26148234 10.1038/onc.2015.264

[84] Zhang S , Hua Z, Ba G, et al Antitumor effects of the small molecule DMAMCL in neuroblastoma via suppressing aerobic glycolysis and targeting PFKL. Cancer Cell Int. 2021;21(1):619. 34819091 10.1186/s12935-021-02330-y PMC8613996

[85] Levy AG , Zage PE, Akers LJ, et al The combination of the novel glycolysis inhibitor 3-BrOP and rapamycin is effective against neuroblastoma. Invest New Drugs. 2012;30(1):191–199. 20890785 10.1007/s10637-010-9551-y PMC4831635

[86] Anderson NM , Qin X, Finan JM, et al Metabolic enzyme DLST promotes tumor aggression and reveals a vulnerability to OXPHOS inhibition in high-risk neuroblastoma. Cancer Res. 2021;81(17):4417–4430. 34233924 10.1158/0008-5472.CAN-20-2153 PMC8577318

[87] Bishayee K , Nazim UM, Kumar V, et al Reversing the HDAC-inhibitor mediated metabolic escape in MYCN-amplified neuroblastoma. Biomed Pharmacother. 2022;150(113032):1–12. 10.1016/j.biopha.2022.113032 35486977

[88] Yue M , Jiang J, Gao P, Liu H, Qing G. Oncogenic MYC activates a feedforward regulatory loop promoting essential amino acid metabolism and tumorigenesis. Cell Rep. 2017;21(13):3819–3832. 29281830 10.1016/j.celrep.2017.12.002

[89] Souba WW. Glutamine and cancer. Ann Surg. 1993;218(6):715–728. 8257221 10.1097/00000658-199312000-00004 PMC1243066

[90] Qing G , Li B, Vu A, et al ATF4 regulates MYC-mediated neuroblastoma cell death upon glutamine deprivation. Cancer Cell. 2012;22(5):631–644. 23153536 10.1016/j.ccr.2012.09.021 PMC3510660

[91] Ren P , Yue M, Xiao D, et al ATF4 and N-Myc coordinate glutamine metabolism in MYCN-amplified neuroblastoma cells through ASCT2 activation. J Pathol. 2015;235(1):90–100. 25142020 10.1002/path.4429

[92] Xiao D , Ren P, Su H, et al Myc promotes glutaminolysis in human neuroblastoma through direct activation of glutaminase 2. Oncotarget. 2015;6(38):40655–40666. 26528759 10.18632/oncotarget.5821 PMC4747359

[93] Tjaden B , Baum K, Marquardt V, et al N-Myc-induced metabolic rewiring creates novel therapeutic vulnerabilities in neuroblastoma. Sci Rep. 2020;10(1):7157. 32346009 10.1038/s41598-020-64040-1 PMC7188804

[94] Xia Y , Ye B, Ding J, et al Metabolic reprogramming by MYCN confers dependence on the serine-glycine-one-carbon biosynthetic pathway. Cancer Res. 2019;79(15):3837–3850. 31088832 10.1158/0008-5472.CAN-18-3541 PMC6679782

[95] Guan J , Li M, Wang Y, et al MTHFD1 regulates the NADPH redox homeostasis in MYCN-amplified neuroblastoma. Cell Death Dis. 2024;15(2):124. 38336749 10.1038/s41419-024-06490-3 PMC10858228

[96] Yu Y , Ding J, Zhu S, et al Therapeutic targeting of both dihydroorotate dehydrogenase and nucleoside transport in MYCN-amplified neuroblastoma. Cell Death Dis. 2021;12(9):821. 34462431 10.1038/s41419-021-04120-w PMC8405683

[97] Olsen TK , Dyberg C, Embaie BT, et al DHODH is an independent prognostic marker and potent therapeutic target in neuroblastoma. JCI Insight. 2022;7(17):e153836. 35943801 10.1172/jci.insight.153836 PMC9798925

[98] Zirath H , Frenzel A, Oliynyk G, et al MYC inhibition induces metabolic changes leading to accumulation of lipid droplets in tumor cells. PNAS. 2013;110(25):10258–10263. 23733953 10.1073/pnas.1222404110 PMC3690852

[99] Tao L , Mohammad MA, Milazzo G, et al MYCN-driven fatty acid uptake is a metabolic vulnerability in neuroblastoma. Nat Commun. 2022;13(1):3728. 35764645 10.1038/s41467-022-31331-2 PMC9240069

[100] Röhrig F , Schulze A. The multifaceted roles of fatty acid synthesis in cancer. Nat Rev Cancer. 2016;16(11):732–749. 27658529 10.1038/nrc.2016.89

[101] Ruiz-Pérez MV , Sainero-Alcolado L, Oliynyk G, et al Inhibition of fatty acid synthesis induces differentiation and reduces tumor burden in childhood neuroblastoma. iScience. 2021;24(2):102128. 33659885 10.1016/j.isci.2021.102128 PMC7895756

[102] Méndez-Lucas A , Lin W, Driscoll PC, et al Identifying strategies to target the metabolic flexibility of tumours. Nat Metab. 2020;2(4):335–350. 32694609 10.1038/s42255-020-0195-8 PMC7436715

[103] Sainero-Alcolado L , Liaño-Pons J, Ruiz-Pérez MV, Arsenian-Henriksson M. Targeting mitochondrial metabolism for precision medicine in cancer. Cell Death Differ. 2022;29(7):1304–1317. 35831624 10.1038/s41418-022-01022-y PMC9287557

[104] Hsieh J , Nakashima K, Kuwabara T, Mejia E, Gage FH. Histone deacetylase inhibition-mediated neuronal differentiation of multipotent adult neural progenitor cells. Proc Natl Acad Sci USA. 2004;101(47):16659–16664. 15537713 10.1073/pnas.0407643101 PMC527137

[105] Alborzinia H , Flórez AF, Kreth S, et al MYCN mediates cysteine addiction and sensitizes neuroblastoma to ferroptosis. Nat Cancer. 2022;3(4):471–485. 35484422 10.1038/s43018-022-00355-4 PMC9050595

[106] Anderson CP , Matthay KK, Perentesis JP, et al Pilot study of intravenous melphalan combined with continuous infusion L-S,R-buthionine sulfoximine for children with recurrent neuroblastoma. Pediatr Blood Cancer. 2015;62(10):1739–1746. 26153194 10.1002/pbc.25594

[107] Freitas FP , Alborzinia H, dos Santos AF, et al 7-Dehydrocholesterol is an endogenous suppressor of ferroptosis. Nature. 2024;626(7998):401–410. 38297129 10.1038/s41586-023-06878-9

[108] Ponzoni M , Bachetti T, Corrias MV, et al Recent advances in the developmental origin of neuroblastoma: an overview. J Exp Clin Cancer Res. 2022;41(1):92. 35277192 10.1186/s13046-022-02281-w PMC8915499

[109] Mabe NW , Huang M, Dalton GN, et al Transition to a mesenchymal state in neuroblastoma confers resistance to anti-GD2 antibody via reduced expression of ST8SIA1. Nat Cancer. 2022;3(8):976–993. 35817829 10.1038/s43018-022-00405-x PMC10071839

[110] Zimmerman MW , Durbin AD, He S, et al Retinoic acid rewires the adrenergic core regulatory circuitry of childhood neuroblastoma. Sci Adv. 2024;7(43):eabe0834. 10.1126/sciadv.abe0834 PMC8528416 34669465

[111] Ferguson KM , Gillen SL, Chaytor L, et al Palbociclib releases the latent differentiation capacity of neuroblastoma cells. Dev Cell. 2023;58(19):1967–1982.e8. 37734383 10.1016/j.devcel.2023.08.028 PMC7618569

[112] Leung TCN , Lu SN, Chu CN, et al Temporal quantitative proteomic and phosphoproteomic profiling of SH-SY5Y and IMR-32 neuroblastoma cells during all-trans-retinoic acid-induced neuronal differentiation. Int J Mol Sci . 2024;25(2):1047. 38256121 10.3390/ijms25021047 PMC10816102

[113] Veal GJ , Cole M, Errington J, et al; UKCCSG Pharmacology Working Group. Pharmacokinetics and metabolism of 13-cis-retinoic acid (isotretinoin) in children with high-risk neuroblastoma – a study of the United Kingdom Children’s Cancer Study Group. Br J Cancer. 2007;96(3):424–431. 17224928 10.1038/sj.bjc.6603554 PMC2360017

[114] Ponthan F , Borgström P, Hassan M, et al The vitamin A analogues: 13-cis retinoic acid, 9-cis retinoic acid, and Ro 13-6307 inhibit neuroblastoma tumour growth in vivo. Med Pediatr Oncol. 2001;36(1):127–131. 11464864 10.1002/1096-911X(20010101)36:1<127::AID-MPO1030>3.0.CO;2-B

[115] Bayeva N , Coll E, Piskareva O. Differentiating neuroblastoma: a systematic review of the retinoic acid, its derivatives, and synergistic interactions. J Pers Med. 2021;11(3):211. 33809565 10.3390/jpm11030211 PMC7999600

[116] Matthay KK , Reynolds CP, Seeger RC, et al Long-term results for children with high-risk neuroblastoma treated on a randomized trial of myeloablative therapy followed by 13-cis-retinoic acid: a children’s oncology group study. J Clin Oncol. 2009;27(7):1007–1013. 19171716 10.1200/JCO.2007.13.8925 PMC2738615

[117] Dobrotkova V , Chlapek P, Mazanek P, Sterba J, Veselska R. Traffic lights for retinoids in oncology: molecular markers of retinoid resistance and sensitivity and their use in the management of cancer differentiation therapy. BMC Cancer. 2018;18(1):1059. 30384831 10.1186/s12885-018-4966-5 PMC6211450

[118] Sainero-Alcolado L , Mushtaq M, Liaño-Pons J, et al Expression and activation of nuclear hormone receptors result in neuronal differentiation and favorable prognosis in neuroblastoma. J Exp Clin Cancer Res. 2022;41(1):226. 35850708 10.1186/s13046-022-02399-x PMC9295514

[119] Kohler JA , Imeson J, Ellershaw C, Lie SO. A randomized trial of 13-Cis retinoic acid in children with advanced neuroblastoma after high-dose therapy. Br J Cancer. 2000;83(9):1124–1127. 11027423 10.1054/bjoc.2000.1425 PMC2363577

[120] Giuli MV , Hanieh PN, Giuliani E, et al Current trends in ATRA delivery for cancer therapy. Pharmaceutics. 2020;12(8):707. 32731612 10.3390/pharmaceutics12080707 PMC7465813

[121] Thiele CJ , Reynolds CP, Israel MA. Decreased expression of N-myc precedes retinoic acid-induced morphological differentiation of human neuroblastoma. Nature. 1985;313(6001):404–406. 3855502 10.1038/313404a0

[122] Puissant A , Frumm SM, Alexe G, et al Targeting MYCN in neuroblastoma by BET Bromodomain inhibition. Cancer Discov. 2013;3(3):308–323. 23430699 10.1158/2159-8290.CD-12-0418 PMC3672953

[123] Lee S , Rellinger EJ, Kim KW, et al Bromodomain and extraterminal inhibition blocks tumor progression and promotes differentiation in neuroblastoma. Surgery. 2015;158(3):819–826. 26067464 10.1016/j.surg.2015.04.017 PMC4536146

[124] Sun Y , Han J, Wang Z, et al Safety and efficacy of bromodomain and extra-terminal inhibitors for the treatment of hematological malignancies and solid tumors: a systematic study of clinical trials. Front Pharmacol. 2021;11(621093)::1–15. 10.3389/fphar.2020.621093 PMC7870522 33574760

[125] Savino M , Annibali D, Carucci N, et al The action mechanism of the Myc inhibitor termed Omomyc may give clues on how to target Myc for cancer therapy. PLoS One. 2011;6(7):e22284. 21811581 10.1371/journal.pone.0022284 PMC3141027

[126] Beaulieu ME , Jauset T, Massó-Vallés D, et al Intrinsic cell-penetrating activity propels Omomyc from proof of concept to viable anti-MYC therapy. Sci Transl Med. 2019;11(484):eaar5012. 30894502 10.1126/scitranslmed.aar5012 PMC6522349

[127] Müller I , Larsson K, Frenzel A, et al Targeting of the MYCN protein with small molecule c-MYC inhibitors. PLoS One. 2014;9(5):e97285. 24859015 10.1371/journal.pone.0097285 PMC4032254

[128] Lampis S , Raieli S, Montemurro L, et al The MYCN inhibitor BGA002 restores the retinoic acid response leading to differentiation or apoptosis by the mTOR block in MYCN-amplified neuroblastoma. J Exp Clin Cancer Res. 2022;41(1):160. 35490242 10.1186/s13046-022-02367-5 PMC9055702

[129] Dzieran J , Garcia AR, Westermark UK, et al MYCN-amplified neuroblastoma maintains an aggressive and undifferentiated phenotype by deregulation of estrogen and NGF signaling. Proc Natl Acad Sci U S A. 2018;115(6):E1229–E1238. 29374092 10.1073/pnas.1710901115 PMC5819392

[130] Ribeiro D , Klarqvist MDR, Westermark UK, et al Regulation of nuclear hormone receptors by MYCN-driven mirnas impacts neural differentiation and survival in neuroblastoma patients. Cell Rep. 2016;16(4):979–993. 27396325 10.1016/j.celrep.2016.06.052

[131] Lovén J , Zinin N, Wahlström T, et al MYCN-regulated microRNAs repress estrogen receptor-α (ESR1) expression and neuronal differentiation in human neuroblastoma. Proc Natl Acad Sci U S A. 2010;107(4):1553–1558. 20080637 10.1073/pnas.0913517107 PMC2824410

[132] Phimmachanh M , Han JZR, O’Donnell YEI, Latham SL, Croucher DR. Histone deacetylases and histone deacetylase inhibitors in neuroblastoma. Front Cell Dev Biol. 2020;8(578770):1–14. 33117806 10.3389/fcell.2020.578770 PMC7575710

[133] Van Tilburg CM , Witt R, Heiss M, et al INFORM2 NivEnt: The first trial of the INFORM2 biomarker driven phase I/II trial series: The combination of nivolumab and entinostat in children and adolescents with refractory high-risk malignancies. BMC Cancer. 2020;20(1):523. 32503469 10.1186/s12885-020-07008-8 PMC7275428

[134] Zhang Y , Wang X, Mu Q, et al Histone H3 acetylation is involved in retinoid acid-induced neural differentiation through increasing mitochondrial function. Biomedicines. 2023;11(12):3251. 38137472 10.3390/biomedicines11123251 PMC10741432

[135] Jiang H , Greathouse RL, Tiche SJ, et al Mitochondrial uncoupling induces epigenome remodeling and promotes differentiation in neuroblastoma. Cancer Res. 2023;83(2):181–194. 36318118 10.1158/0008-5472.CAN-22-1029 PMC9851961

[136] Yang Y , Wang S, Cai J, et al Targeting ARHGEF12 promotes neuroblastoma differentiation, MYCN degradation, and reduces tumorigenicity. Cell Oncol (Dordr). 2023;46(1):133–143. 36520365 10.1007/s13402-022-00739-9 PMC12974740

[137] Pruss M , Dwucet A, Tanriover M, et al Dual metabolic reprogramming by ONC201/TIC10 and 2-Deoxyglucose induces energy depletion and synergistic anti-cancer activity in glioblastoma. Br J Cancer. 2020;122(8):1146–1157. 32115576 10.1038/s41416-020-0759-0 PMC7156767

[138] Wu JC , Huang CC, Wang PW, et al ONC201 suppresses neuroblastoma growth by interrupting mitochondrial function and reactivating nuclear ATRX expression while decreasing MYCN. Int J Mol Sci . 2023;24(2):1649. 36675163 10.3390/ijms24021649 PMC9867473

[139] Gardner SL , Tarapore RS, Allen J, et al Phase I dose escalation and expansion trial of single agent ONC201 in pediatric diffuse midline gliomas following radiotherapy. Neurooncol. Adv. 2022;4(1):vdac143. 36382108 10.1093/noajnl/vdac143 PMC9639395

[140] Schulz G , Cheresh DA, Varki NM, et al Detection of ganglioside GD2 in tumor tissues and sera of neuroblastoma patients. Cancer Res. 1984;44(12 Pt 1):5914–5920. 6498849

[141] Yu AL , Gilman AL, Ozkaynak MF, et al; Children's Oncology Group. Anti-GD2 antibody with GM-CSF, interleukin-2, and isotretinoin for neuroblastoma. N Engl J Med. 2010;363(14):1324–1334. 20879881 10.1056/NEJMoa0911123 PMC3086629

[142] Kushner BH , Cheung IY, Modak S, et al Humanized 3F8 Anti-GD2 monoclonal antibody dosing with granulocyte-macrophage colony-stimulating factor in patients with resistant neuroblastoma: a phase 1 clinical trial. JAMA Oncol. 2018;4(12):1729–1735. 30326045 10.1001/jamaoncol.2018.4005 PMC6440722

[143] Furman WL , Federico SM, McCarville MB, et al A Phase II Trial of Hu14.18K322A in combination with induction chemotherapy in children with newly diagnosed high-risk neuroblastoma. Clin Cancer Res. 2019;25(21):6320–6328. 31601569 10.1158/1078-0432.CCR-19-1452 PMC6825564

[144] Cheung IY , Cheung NV, Modak S, et al Survival impact of anti-GD2 antibody response in a phase II ganglioside vaccine trial among patients with high-risk neuroblastoma with prior disease progression. J Clin Oncol. 2020;39(3):215–226. 33326254 10.1200/JCO.20.01892 PMC8253584

[145] Cheung IY , Mauguen A, Modak S, et al Effect of oral β-glucan on antibody response to ganglioside vaccine in patients with high-risk neuroblastoma: a phase 2 randomized clinical trial. JAMA Oncol. 2023;9(2):242–250. doi: https://doi.org/10.1001/jamaoncol.2022.5999 36547975 PMC9936346

[146] Kramer K , Pandit-Taskar N, Kushner BH, et al Phase 1 study of intraventricular 131I-omburtamab targeting B7H3 (CD276)-expressing CNS malignancies. J Hematol Oncol. 2022;15(1):165. 36371226 10.1186/s13045-022-01383-4 PMC9655863

[147] Espinosa-Cotton M , Cheung NKV. Bispecific antibodies for the treatment of neuroblastoma. Pharmacol Ther. 2022;237(108241):1–9. 10.1016/j.pharmthera.2022.108241 PMC10351215 35830901

[148] Slyper M , Porter CBM, Ashenberg O, et al A single-cell and single-nucleus RNA-Seq toolbox for fresh and frozen human tumors. Nat Med. 2020;26(5):792–802. 32405060 10.1038/s41591-020-0844-1 PMC7220853

[149] Wölfl M , Jungbluth AA, Garrido F, et al Expression of MHC class I, MHC class II, and cancer germline antigens in neuroblastoma. Cancer Immunol Immunother. 2005;54(4):400–406. 15449039 10.1007/s00262-004-0603-z PMC11034322

[150] Richards RM , Sotillo E, Majzner RG. CAR T Cell Therapy for Neuroblastoma. Front Immunol. 2018;9(2380):1–15. 30459759 10.3389/fimmu.2018.02380 PMC6232778

[151] ClinicalTrials.gov. Bethesda (MD). GD2 specific CAR and interleukin-15 expressing autologous NKT cells to treat children with neuroblastoma (GINAKIT2). Published 2017. https://clinicaltrials.gov/ct2/show/NCT03294954

[152] ClinicalTrials.gov. Bethesda (MD). Anti-GD2 CAR T cells in pediatric patients affected by high risk and/or relapsed/refractory neuroblastoma or other GD2-positive solid tumors. Published 2017. https://clinicaltrials.gov/ct2/show/NCT03373097

[153] Del Bufalo F , De Angelis B, Caruana I, et al; Precision Medicine Team–IRCCS Ospedale Pediatrico Bambino Gesù. GD2-CART01 for relapsed or refractory high-risk neuroblastoma. N Engl J Med. 2023;388(14):1284–1295. 37018492 10.1056/NEJMoa2210859

[154] Bosse KR , Raman P, Zhu Z, et al Identification of GPC2 as an oncoprotein and candidate immunotherapeutic target in high-risk neuroblastoma. Cancer Cell. 2017;32(3):295–309.e12. 28898695 10.1016/j.ccell.2017.08.003 PMC5600520

[155] Li N , Torres MB, Spetz MR, et al CAR T cells targeting tumor-associated exons of glypican 2 regress neuroblastoma in mice. Cell Rep Med. 2021;2(6):1–16. 10.1016/j.xcrm.2021.100297 PMC8233664 34195677

[156] Yarmarkovich M , Marshall QF, Warrington JM, et al Targeting of intracellular oncoproteins with peptide-centric CARs. Nature. 2023;623(7988):820–827. 37938771 10.1038/s41586-023-06706-0 PMC10665195

[157] Moghimi B , Muthugounder S, Jambon S, et al Preclinical assessment of the efficacy and specificity of GD2-B7H3 SynNotch CAR-T in metastatic neuroblastoma. Nat Commun. 2021;12(1):511. 33479234 10.1038/s41467-020-20785-x PMC7820416

[158] Mody R , Yu AL, Naranjo A, et al Irinotecan, temozolomide, and dinutuximab with GM-CSF in children with refractory or relapsed neuroblastoma: a report from the children’s oncology group. J Clin Oncol. 2020;38(19):2160–2169. 32343642 10.1200/JCO.20.00203 PMC7325366

[159] Davis KL , Fox E, Merchant MS, et al Nivolumab in children and young adults with relapsed or refractory solid tumours or lymphoma (ADVL1412): a multicentre, open-label, single-arm, phase 1-2 trial. Lancet Oncol. 2020;21(4):541–550. 32192573 10.1016/S1470-2045(20)30023-1 PMC7255545

[160] ClinicalTrials.gov. Bethesda (MD). Testing the combination of two immunotherapy drugs (Nivolumab and Ipilimumab) in children, adolescent, and young adult patients with relapsed/refractory cancers that have an increased number of genetic changes, the 3ci study. Published online 2020.

[161] ClinicalTrials.gov. Bethesda (MD). Nivolumab with or without ipilimumab in treating younger patients with recurrent or refractory solid tumors or sarcomas. Published online 2014.

[162] Sengupta S , Das S, Crespo AC, et al Mesenchymal and adrenergic cell lineage states in neuroblastoma possess distinct immunogenic phenotypes. Nat Cancer. 2022;3:1228–1246. doi: https://doi.org/10.1038/s43018-022-00427-5 36138189 PMC10171398

[163] Bottino C , Dondero A, Bellora F, et al Natural killer cells and neuroblastoma: tumor recognition, escape mechanisms, and possible novel immunotherapeutic approaches. Front Immunol. 2014;5(56):1–11. 24575100 10.3389/fimmu.2014.00056 PMC3921882

[164] Brandetti E , Veneziani I, Melaiu O, et al MYCN is an immunosuppressive oncogene dampening the expression of ligands for NK-cell-activating receptors in human high-risk neuroblastoma. Oncoimmunology. 2017;6(6):e1316439. 28680748 10.1080/2162402X.2017.1316439 PMC5486189

[165] Modak S , Le Luduec JB, Cheung IY, et al Adoptive immunotherapy with haploidentical natural killer cells and Anti-GD2 monoclonal antibody m3F8 for resistant neuroblastoma: results of a phase I study. Oncoimmunology. 2018;7(8):e1461305. 30221057 10.1080/2162402X.2018.1461305 PMC6136849

[166] Wienke J , Visser LL, Kholosy WM, et al Integrative analysis of neuroblastoma by single-cell RNA sequencing identifies the NECTIN2-TIGIT axis as a target for immunotherapy. Cancer Cell. 2024;42(2):283–300.e8. 38181797 10.1016/j.ccell.2023.12.008 PMC10864003

