# Erratum to: Defining neuroblastoma: from origin to precision medicine

**DOI:** 10.1093/neuonc/noae218

**Published:** 2024-10-26

This is an erratum to: Lourdes Sainero-Alcolado, Tomas Sjöberg Bexelius, Giuseppe Santopolo, Ye Yuan, Judit Liaño-Pons, Marie Arsenian-Henriksson, Defining neuroblastoma: from origin to precision medicine, *Neuro-Oncology*, 2024;, noae152, https://doi.org/10.1093/neuonc/noae152

In the originally published version of this paper, there was an error in the references list. Reference 145 was incorrectly given as:

Sengupta S, Das S, Crespo AC, et al.. Mesenchymal and adrenergic cell lineage states in neuroblastoma possess distinct immunogenic phenotypes. *Nat Cancer*. 2022;3:1228–1246. doi:10.1038/s43018-022-00427-5

This reference should have been number 162. Reference 145 should have been:

Cheung IY, Mauguen A, Modak S, et al. Effect of oral β-glucan on antibody response to ganglioside vaccine in patients with high-risk neuroblastoma: a phase 2 randomized clinical trial. *JAMA Oncol*. 2023;9(2):242–250. doi:10.1001/jamaoncol.2022.5999

This error, for which the publisher apologizes, has been corrected. The references from 162 onwards have also been renumbered to account for this error.

