# Supplementary material for: Defining neuroblastoma: From origin to precision medicine
Source: Neuro Oncol. 2024 Aug 5;26(12):2174–92. doi: 10.1093/neuonc/noae152 (PMC11630532; doi:10.1093/neuonc/noae152)
Supplement: noae152_suppl_Supplementary_Material [file noae152_suppl_supplementary_material.docx]

**Supplementary Material**

**Defining Neuroblastoma: from Origin to Precision Medicine**

Lourdes Sainero-Alcolado^1^, Tomas Sjöberg Bexelius^2,3^, Giuseppe Santopolo^1^, Ye Yuan^1^, Judit Liaño-Pons^1,^*, and Marie Arsenian-Henriksson^1, 4^*

^1^Department of Microbiology, Tumor and Cell Biology (MTC), Biomedicum B7, Karolinska Institutet, SE-17165, Stockholm, Sweden.

^2^Paediatric Oncology Unit, Astrid Lindgren's Children Hospital, SE-171 64 Solna, Sweden.

^3^Department of Women's and Children's Health, Karolinska Institutet, SE-171 77 Stockholm, Sweden.

^4^Lund University, Division of Translational Cancer Research, Department of Laboratory Medicine, Lund, SE-223 81, Sweden.

*Corresponding authors: judit.liano.pons@ki.se; marie.arsenian.henriksson@ki.se

The authors declare no potential conflicts of interest.

Content:

Tables S1-4

Supplementary references

**Table S1. Genetic Syndromes Associated with Neuroblastoma**. Inheritance types, clinical manifestations, associated gene alterations, and references are listed.

| **Syndrome** | **Type** | **Clinical manifestations** | **Gene** | **Genetic alterations** | **References** |
| --- | --- | --- | --- | --- | --- |
| Congenital Central Hypoventilation Syndrome (CCHS) | Autosomal dominant | Insufficient response to hypercapnia and hypoxia, especially during sleep | *PHOX2B* | Polyalanine repeat mutations (90%) / frameshift, missense, and nonsense mutations (10%) | ^1^ |
| Beckwith-Wiedemann Syndrome (BWS) | Autosomal dominant / congenital imprinting | Overgrowth and increased childhood cancer risk | *IGF2, H19, CDKN1C, KCNQ1051* | Abnormal methylation in genes in chr 11p15.5 / pathogenic variants | ^2,3^ |
| Costello Syndrome | Autosomal dominant | Severe postnatal failure to thrive, short stature, intellectual disability, and increased risk for cancer | *HRAS* | Germline pathogenic variants | ^4,5^ |
| Fanconi Anemia (FA) | Autosomal recessive | Risk of congenital malformations, bone marrow failure and cancer | *FANCD1/BRCA2, FANCN, FANCA, FANCB, FANCC, FANCD2, FANCE, FANCF, FANCG, FANCI, FANCJ* | Germline mutations | ^6–8^ |
| Li Fraumeni Syndrome (LFS) | Autosomal dominant | Higher risk of cancer | *TP53* | Germline pathogenic variants | ^9^ |
| Neurofibromatosis type 1 (NF1) Syndrome | Autosomal dominant | Café-au-lait macules, seizures, chronic pain, vascular issues, bone defects, central and peripheral nervous system tumors, breast cancer, and other types | *NF1* | Deletions / pathogenic variants | ^10–12^ |
| Hirschsprung Disease (HSCR) | Multigenic congenital disease | Affecting the enteric nervous system, leading to functional intestinal obstruction | *RET* | Loss-of-function mutations in the coding region | ^7,8^ |
|  |  |  | *EDNRB* | Mutations |  |
|  |  |  | *PHOX2B* | Polymorphisms / Deletion 4p12 |  |
| Noonan Syndrome | Autosomal dominant | Short stature, webbing of the neck, hypertelorism, cryptorchidism, feeding difficulties, and mild mental retardation | *PTPN11* | Gain of function mutations | ^13,14^ |
| Familial  paraganglioma/pheochromocytoma (PGL/PCC) | Autosomal dominant | Tumors in the paraganglia of the peripheral nervous system | *SDHB, SDHD, SDHC, SDHA, SDHAF2, MAX, TMEM127* | Heterozygous germline mutations | ^15^ |
| Weaver Syndrome | Autosomal dominant | Overgrowth, macrocephaly, accelerated bone age, intellectual disability, higher risk of cancer | *EZH2* | *De novo* mutations, *e.g.* *p.(Ala682Thr)* and *p.(Glu745Lys)* | ^16^ |

**Table S2.** **The International Neuroblastoma Staging System (INSS)**. This staging was used prior to the

implementation of the more complete INRGSS system.

| **Stage** | **Definition** |
| --- | --- |
| Stage 1 | Tumors that can be completely removed surgically. The lymph nodes attached to the tumor may or may not contain cancer cells, but the lymph nodes near the tumor are clear. |
| Stage 2A | Tumors located only in the primary site and cannot be completely removed during surgery. Nearby lymph nodes do not contain cancer cells. |
| Stage 2B | Tumors located only in the tissue area where the disease started and may or may not be completely removed by surgery. Nearby lymph nodes do contain cancer cells. |
| Stage 3 | Tumors that cannot be removed with surgery alone. The disease has spread only to nearby lymph nodes and/or other areas. |
| Stage 4 | Tumors that have spread to distant lymph nodes, bones, bone marrow, liver, skin, and/or other organs (except for skin, liver, and/or bone marrow in infants younger than 1). |
| Stage 4S | Tumors located in the primary site and which have spread only to the skin, liver, and/or bone marrow in infants younger than 12 months. For this stage, the spread of the cancer to the bone marrow is usually less than 10% of cells examined. |

**Table S3. Classification of NB stages according to INRGSS.**

| **INRG stage** | **Age at diagnosis (months)** | **Histologic category** | **Grade of tumor differentiation** | ***MYCN-* amplification** | **11q aberration** | **Ploidy** | **Pre-treatment risk group** |
| --- | --- | --- | --- | --- | --- | --- | --- |
| L1/L2 |  | GN maturing; GNB intermixed |  |  |  |  | A = very low |
| L1 |  | Any except GN maturing or GNB intermixed |  | Non-amplified |  |  | B = very low |
|  |  |  |  | Amplified |  |  | K = high |
| L2 | <18 | Any except GN maturing or GNB intermixed |  | Non-amplified | None |  | D = low |
|  |  |  |  |  | Yes |  | G = intermediate |
|  | ≥ 18 | GNB nodular; neuroblastoma | Differentiating | Non-amplified | None |  | E = low |
|  |  |  |  |  | Yes |  | H = intermediate |
|  |  |  | Poorly differentiated or undifferentiated |  |  |  |  |
|  |  |  |  | Amplified |  |  | N = high |
| M | <18 |  |  | Non-amplified |  | Hyper-diploid | F = low |
|  | <12 |  |  | Non-amplified |  | Diploid | I = intermediate |
|  | 12 to <18 |  |  | Non- amplified |  | Diploid | J = intermediate |
|  | <18 |  |  | Amplified |  |  | O = high |
|  | ≥ 18 |  |  |  |  |  | P = high |
| MS | <18 |  |  | Non-amplified | None |  | C = very low |
|  |  |  |  |  | Yes |  | Q = high |
|  |  |  |  | Amplified |  |  | R = high |

**Table S4. Overview of a Selection of Neuroblastoma Models. (A)** 2D cell culture; **(B)** 3D spheres and organoids; and **(C)** animal models.

1. **2D Models:** selection of human NB cell lines, origin, genetic features, type (N/S/ I and MES/ADRN), and references.

ND=not defined; MNA=*MYCN*-amplified; WT=wildtype; del=deletion; mut=mutation; N= neuroblastic; S= substrate adherent; I=intermediate; MES=mesenchymal; ADRN=adrenergic.

| Cell line | Patient information | | | | | Genetic features | | | | | | | Type | | References |
| --- | --- | --- | --- | --- | --- | --- | --- | --- | --- | --- | --- | --- | --- | --- | --- |
|  | **Patient (age/**  **gender)** | **Primary tumor** | **Established from** | **Treatment** | **Stage** | ***MYCN*** | ***ALK*** | ***TP53*** | **Other (1p, 17q, 11q)** | | | | **N, S, I** | **MES, ADRN** |  |
| NB-69 | 1.4/Male | ND | Bone marrow | No | 3 | Non-MNA | ND | WT | ND | | | | ND | ADRN | ^17,18^ |
| NBL-S | 3.6/Male | Adrenal gland | Adrenal gland | No | 3 | Non-MNA | ND | WT | 11q del | | | | ND | ADRN | ^17–20^ |
| SK-N-FI | 11/Male | ND | Bone marrow | Yes | 4 | Non-MNA | WT | Mut M246R | *NF1* mut | | | | N | ADRN | ^18,21^ |
| SK-N-SH | 4/Female | Thorax | Bone marrow | Yes | 4 | Non-MNA | F1174 L | WT | 1q trisomy  17q gain | | | | I | ADRN/MES | ^19,22,23^ |
| SH-EP |  |  |  |  |  |  |  |  |  |  |  |  | S | MES | ^22^ |
| SH-SY5Y |  |  |  |  |  |  |  |  |  |  |  |  | N | ADRN | ^20,24,25^ |
| SK-N-AS | 8/Female | Adrenal  gland | Bone marrow | Yes | 4 | Non-MNA | WT | Mut H168R | 1 p del, 11q del | | |  | S | MES | ^19,26^ |
| IMR-5 | 1.1/Male | ND | Abdomen | ND | ND | MNA | ND | WT | 11q del |  |  |  | N | ADRN | ^20,27^ |
| IMR32 | 1.1/Male | ND | Abdomen | No | ? | MNA | Amplified | WT | 1p del, 17q gain | | |  | N | ADRN | ^17,20^ |
| KELLY | 1/Female | ND | Brain | ND | ND | MNA | F1174 L | P177T | 11q del |  |  |  | ND | ADRN | ^18,23,26–28^ |
| GI-ME-N | 3.6/Female | Adrenal gland | Bone marrow | Yes | 4 | Non-MNA | ND | ND | 1p del |  |  |  | N | MES | ^29^ |
| LA-N-5 | 4/Male | ND | Bone marrow | No | ND | MNA | R1275Q | WT | ND |  |  |  | ND | ADRN | ^18,22^ |
| NB-1 | 2.3/Male | ND | Cervical lymph node | ND | ND | MNA | WT; Amplified | WT | ND |  |  |  | ND | ND | ^30^ |
| SK-N-BE(1) | 1.1/Male | ND | Bone marrow | No | 4 | MNA | WT | WT | 1p del | | |  | N | ADRN | ^31^ |
| SK-N-BE(2) | 2.2/Male | ND | Bone marrow | Yes | 4 | MNA | WT | Mut C135F | 1p del/LOH  t(3;17)(p21;q21) | | |  | N | ADRN | ^20,24^ |
| SK-N-DZ | 2/Female | ND | Bone marrow | ND | ND | MNA | WT | R110L | 11q del |  |  |  | ND | ADRN | ^23,26^ |
| SMS-KAN | 3/Female | Pelvic | Bone marrow/  lymph node | Yes | 4 | MNA | ND | WT | 1p del  Del(17);t(17;18) | | |  | N | ADRN | ^32,33^ |
| SMS-KCN69n | 0.11/Male | Adrenal gland | Bone  marrow/  lymph node | No | 4 | MNA | R1275Q | WT | 1p del  17q gain | | |  | N | ADRN | ^24^ |

1. **3D Models.** Summary of 3D systems with higher complexity than cell culture and that recreate the structure of the tissue of origin. PDX=patient-derived xenograft.

| Type | Description | References |
| --- | --- | --- |
| Multicellular tumor spheroids (MCTs) | NB cell lines grown in low adherent conditions | ^34,35^ |
| Patient-derived spheres | Spheres established directly from patient tissue | ^34,36^ |
| Patient-derived organoids | Derived from:   - wild-type cell lines from human pluripotent stem cells (hPSCs) - induced pluripotent stem cells (iPSCs) derived from patients - directly from patient tissue | ^34,36,37^ |
| PDX-derived organoids | *In vitro* culture of *in vivo* expanded patient tissue | ^34,36^ |

1. **Main Animal Models in Mouse, Zebrafish, and Chicken.** Genetic alterations, penetrance, and references. NB cell lines and patient-derived xenografts are also listed. ND=not defined. PDX=patient-derived xenograft.

| Organism | Model Name | Modelled Genetic alteration | Penetrance | Comments | References |
| --- | --- | --- | --- | --- | --- |
| Mouse | TH-*MYCN* | *MYCN*-amplification | 5-100 | - | ^38,39^ |
|  | TH-*ALKF1174L*/*MYCN* | *MYCN*-amplification + *ALK* mutation | 100 | - | ^39^ |
|  | TH-*MYCN*/*CASP8*(KO) | *MYCN*-amplification + Caspase 8 deficiency | ND | 37% increase in bone marrow metastasis | ^40^ |
|  | TH-*MYCN*/*Trp53*(KI/Kl) | *MYCN*-amplification + loss of p53 | 75% | Resistance to iodizing radiation | ^41^ |
|  | LSL-*MYCN*; Dbh:iCre | *MYCN*-amplification | > 75% | - | ^42^ |
|  | Dbh-*iCre*/CAG-LSL-*ALKF1174L* | *ALK* mutation | < 50% | - | ^43^ |
|  | TRE-*SV40* Tag | *MYCN* overexpression | ND | Bilateral large adrenal tumors | ^44^ |
|  | NB cell line xenografts | *Depending on the cell line* | ND | Different outcomes depending on the cell line used | ^45^ |
|  | Patient-derived xenografts (PDX) | *Depending on the tumor* | ND | Different outcomes depending on the patient tumor used | ^46,47^ |
| Zebrafish | Tg (dβh:*EGFP*-*MYCN*)  Tg(dβh:*MYCN*) | *MYCN*-amplification | 20-30% | - | ^48,49^ |
|  | Tg(dβh:*MYC*) | *MYC* overexpression | 80-100% | - | ^49^ |
|  | Tg(dβh:*EGFP*; dβh:*ALKF1174L*) | *ALK* mutation | ND | - | ^32^ |
|  | Tg(dβh:*EGFP-MYCN*; dβh-*LMO1*) | *MYCN*-amplification + *LMO1* overexpression | 80% | - | ^50^ |
|  | Tg (dβh:EGFP-*MYCN*; *nf1a^-/-^nf1b^+/-^ // nf1a^-/-^; nf1b^+/+^*) | *MYCN*-amplification + *NF1* knockout | 60-80% | NB with mutations affecting the RAS-MAPK pathway | ^51^ |
|  | Tg(dβh:ptpn11mut-EGFP);  (dβh:*MYCN*) | *MYCN*-amplification + mutant *ptpn11* | 75% | ptpn11^E69K^ | ^52^ |
|  | Tg(dβh:Gab2wt;dβh:EGFP); (dβh:MYCN) | *MYCN*-amplification + *GAB2* overexpression | 90% | Activation of the SHP2-RAS-ERK pathway | ^52^ |
|  | Tg(dβh:*EGFP*;dβh:*LIN28B*_WT); (dβh:*EGFP-MYCN*) | *MYCN*-amplification + overexpression of LIN28B | 50% | - | ^53^ |
|  | Tg(dβh:*EGFP;dβh:LIN28B*_MU); (dβh:*EGFP-MYCN*) | *MYCN*-amplification + overexpression of mutant LIN28B | 60% | - | ^53^ |
| Chicken | Chick chorioallantoic  membrane (CAM) xenograft | Depending on cells or tumor material used | ND | Different outcomes depending on the use of cell lines or tumor-derived material | ^54^ |

**Supplementary references**

1. Windels ML, Cordier F, Van Dorpe J, Ferdinande L, Creytens D. PHOX2B: a diagnostic cornerstone in neurocristopathies and neuroblastomas. *J Clin Pathol*. 2024;77(6):378. doi:10.1136/jcp-2023-209047

2. Cöktü S, Spix C, Kaiser M, et al. Cancer incidence and spectrum among children with genetically confirmed Beckwith-Wiedemann spectrum in Germany: a retrospective cohort study. *Br J Cancer*. 2020;123(4):619-623. doi:10.1038/s41416-020-0911-x

3. Emery LG, Shields M, Shah NR, Garbes A. Neuroblastoma associated with beckwith-wiedemann syndrome. *Cancer*. 1983;52(1):176-179. doi:https://doi.org/10.1002/1097-0142(19830701)52:1<176::AID-CNCR2820520132>3.0.CO;2-V

4. Astiazaran-Symonds E, Ney GM, Higgs C, et al. Cancer in Costello syndrome: a systematic review and meta-analysis. *Br J Cancer*. 2023;128(11):2089-2096. doi:10.1038/s41416-023-02229-7

5. Gripp KW. Tumor predisposition in Costello syndrome. *Am J Med Genet C Semin Med Genet*. 2005;137C(1):72-77. doi:https://doi.org/10.1002/ajmg.c.30065

6. Nalepa G, Clapp DW. Fanconi anaemia and cancer: an intricate relationship. *Nat Rev Cancer*. 2018;18(3):168-185. doi:10.1038/nrc.2017.116

7. Bissig H, Staehelin F, Tolnay M, et al. Co-occurrence of neuroblastoma and nephroblastoma in an infant with Fanconi’s anemia. *Hum Pathol*. 2002;33(10):1047-1051. doi:https://doi.org/10.1053/hupa.2002.128062

8. McReynolds LJ, Biswas K, Giri N, Sharan SK, Alter BP. Genotype-cancer association in patients with Fanconi anemia due to pathogenic variants in FANCD1 (BRCA2) or FANCN (PALB2). *Cancer Genet*. 2021;258-259:101-109. doi:https://doi.org/10.1016/j.cancergen.2021.10.001

9. Kumamoto T, Yamazaki F, Nakano Y, et al. Medical guidelines for Li–Fraumeni syndrome 2019, version 1.1. *Int J Clin Oncol*. 2021;26(12):2161-2178. doi:10.1007/s10147-021-02011-w

10. Karaconji T, Whist E, Jamieson R V, Flaherty MP, Grigg JRB. Neurofibromatosis Type 1: Review and Update on Emerging Therapies. *Asia-Pacific Journal of Ophthalmology*. 2019;8(1):62-72. doi:https://doi.org/10.22608/APO.2018182

11. Kehrer-Sawatzki H, Cooper DN. Classification of NF1 microdeletions and its importance for establishing genotype/phenotype correlations in patients with NF1 microdeletions. *Hum Genet*. 2021;140(12):1635-1649. doi:10.1007/s00439-021-02363-3

12. Rana S, Low CE, Karthikeyan M, Koh MJA, Ngeow J, Chiang J. A Systematic Review of Diagnostic Modalities and Strategies for the Assessment of Complications in Adult Patients with Neurofibromatosis Type 1. *Cancers (Basel)*. 2024;16(6). doi:10.3390/cancers16061119

13. Cotton JL, Williams RG. Noonan Syndrome and Neuroblastoma. *Arch Pediatr Adolesc Med*. 1995;149(11):1280-1281. doi:10.1001/archpedi.1995.02170240098019

14. Lopez-Miranda B, Westra SJ, Boechat MI, Yazdani S. Noonan syndrome associated  with neuroblastoma: a case report. *Pediatr Radiol*. 1997;27(4):324-326. doi:10.1007/s002470050140

15. Schimke RN, Collins DL, Stolle CA. Paraganglioma, neuroblastoma, and a SDHB mutation: Resolution of a 30-year-old mystery. *Am J Med Genet A*. 2010;152A(6):1531-1535. doi:https://doi.org/10.1002/ajmg.a.33384

16. Cohen ASA, Yap DB, Lewis MES, et al. Weaver Syndrome-Associated EZH2 Protein Variants Show Impaired Histone Methyltransferase Function In Vitro. *Hum Mutat*. 2016;37(3):301-307. doi:10.1002/humu.22946

17. Carr J, Bell E, Pearson ADJ, et al. Increased frequency of aberrations in the p53/MDM2/p14(ARF) pathway in  neuroblastoma cell lines established at relapse. *Cancer Res*. 2006;66(4):2138-2145. doi:10.1158/0008-5472.CAN-05-2623

18. Upton K, Modi A, Patel K, et al. Epigenomic profiling of neuroblastoma cell lines. *Sci Data*. 2020;7(1). doi:10.1038/s41597-020-0458-y

19. Mosse YP, Greshock J, Margolin A, et al. High-resolution detection and mapping of genomic DNA alterations in  neuroblastoma. *Genes Chromosomes Cancer*. 2005;43(4):390-403. doi:10.1002/gcc.20198

20. Sanmartín E, Muñoz L, Piqueras M, et al. Deletion of 11q in Neuroblastomas Drives Sensitivity to PARP Inhibition. *Clin Cancer Res*. 2017;23(22):6875-6887. doi:10.1158/1078-0432.CCR-17-0593

21. Hölzel M, Huang S, Koster J, et al. NF1 is a tumor suppressor in neuroblastoma that determines retinoic acid response  and disease outcome. *Cell*. 2010;142(2):218-229. doi:10.1016/j.cell.2010.06.004

22. Chen Y, Takita J, Choi YL, et al. Oncogenic mutations of ALK kinase in neuroblastoma. *Nature*. 2008;455(7215):971-974. doi:10.1038/nature07399

23. Mossé YP, Laudenslager M, Longo L, et al. Identification of ALK as a major familial neuroblastoma predisposition gene. *Nature*. 2008;455(7215):930-935. doi:10.1038/nature07261

24. Walton JD, Kattan DR, Thomas SK, et al. Characteristics of stem cells from human neuroblastoma cell lines and in tumors. *Neoplasia*. 2004;6(6):838-845. doi:10.1593/neo.04310

25. Xie H rong, Hu L sen, Li G yi. SH-SY5Y human neuroblastoma cell line: in vitro cell model of dopaminergic  neurons in Parkinson’s disease. *Chin Med J (Engl)*. 2010;123(8):1086-1092.

26. Carén H, Kryh H, Nethander M, et al. High-risk neuroblastoma tumors with 11q-deletion display a poor prognostic,  chromosome instability phenotype with later onset. *Proc Natl Acad Sci U S A*. 2010;107(9):4323-4328. doi:10.1073/pnas.0910684107

27. Arnhold V, Schmelz K, Proba J, et al. Reactivating TP53 signaling by the novel MDM2 inhibitor DS-3032b as a therapeutic  option for high-risk neuroblastoma. *Oncotarget*. 2018;9(2):2304-2319. doi:10.18632/oncotarget.23409

28. Harenza JL, Diamond MA, Adams RN, et al. Transcriptomic profiling of 39 commonly-used neuroblastoma cell lines. *Sci Data*. 2017;4:170033. doi:10.1038/sdata.2017.33

29. Donti E, Longo L, Tonini GP, et al. *Cytogenetic and Molecular Study of Two Human Neuroblastoma Cell Lines*.

30. Miyake S, Shimo Y, Kitamura T. [Morphological differentiation in vitro of human continuous and functional  neuroblastoma cell line, NB-I under treatment of (But)2cAMP (author’s transl)]. *No Shinkei Geka*. 1975;3(5):407-414.

31. Biedler JL, Roffler-Tarlov S, Schachner M, Freedman LS. Multiple neurotransmitter synthesis by human neuroblastoma cell lines and clones. *Cancer Res*. 1978;38(11 Pt 1):3751-3757.

32. Walton JD, Kattan DR, Thomas SK, et al. Characteristics of Stem Cells from Human Neuroblastoma Cell Lines and in Tumors. *Neoplasia*. 2004;6(6):838-845. doi:10.1593/neo.04310

33. Reynolds CP, Biedler JL, Spengler BA, et al. Characterization of human neuroblastoma cell lines established before and after  therapy. *J Natl Cancer Inst*. 1986;76(3):375-387.

34. Corallo D, Frabetti S, Candini O, et al. Emerging Neuroblastoma 3D In Vitro Models for Pre-Clinical Assessments. *Front Immunol*. 2020;11:584214. doi:10.3389/fimmu.2020.584214

35. Baek N, Seo OW, Kim M, Hulme J, An SSA. Monitoring the effects of doxorubicin on 3D-spheroid tumor cells in real-time. *Onco Targets Ther*. 2016;9:7207-7218. doi:10.2147/OTT.S112566

36. Aaltonen K, Radke K, Adamska A, Seger A, Mañas A, Bexell D. Patient-derived models: Advanced tools for precision medicine in neuroblastoma. *Front Oncol*. 2022;12:1085270. doi:10.3389/fonc.2022.1085270

37. Smith RC, Tabar V. Constructing and Deconstructing Cancers using Human Pluripotent Stem Cells and  Organoids. *Cell Stem Cell*. 2019;24(1):12-24. doi:10.1016/j.stem.2018.11.012

38. Weiss WA, Aldape K, Mohapatra G, Feuerstein BG, Bishop JM. Targeted expression of MYCN causes neuroblastoma in transgenic mice. *EMBO J*. 1997;16(11):2985-2995. doi:10.1093/emboj/16.11.2985

39. Berry T, Luther W, Bhatnagar N, et al. The ALK(F1174L) mutation potentiates the oncogenic activity of MYCN in  neuroblastoma. *Cancer Cell*. 2012;22(1):117-130. doi:10.1016/j.ccr.2012.06.001

40. Teitz T, Inoue M, Valentine MB, et al. Th-MYCN mice with caspase-8 deficiency develop advanced neuroblastoma with bone  marrow metastasis. *Cancer Res*. 2013;73(13):4086-4097. doi:10.1158/0008-5472.CAN-12-2681

41. Yogev O, Barker K, Sikka A, et al. p53 Loss in MYC-Driven Neuroblastoma Leads to Metabolic Adaptations Supporting  Radioresistance. *Cancer Res*. 2016;76(10):3025-3035. doi:10.1158/0008-5472.CAN-15-1939

42. Althoff K, Beckers A, Bell E, et al. A Cre-conditional MYCN-driven neuroblastoma mouse model as an improved tool for  preclinical studies. *Oncogene*. 2015;34(26):3357-3368. doi:10.1038/onc.2014.269

43. Heukamp LC, Thor T, Schramm A, et al. Targeted expression of mutated ALK induces neuroblastoma in transgenic mice. *Sci Transl Med*. 2012;4(141):141ra91. doi:10.1126/scitranslmed.3003967

44. Iwakura H, Ariyasu H, Kanamoto N, et al. Establishment of a novel neuroblastoma mouse model. *Int J Oncol*. 2008;33(6):1195-1199.

45. Krawczyk E, Kitlí Nska J. Preclinical Models of Neuroblastoma-Current Status and Perspectives. Published online 2023. doi:10.3390/cancers

46. Braekeveldt N, Bexell D. Patient-derived xenografts as preclinical neuroblastoma models. *Cell Tissue Res*. 2018;372(2):233-243. doi:10.1007/s00441-017-2687-8

47. Mañas A, Aaltonen K, Andersson N, et al. Clinically relevant treatment of PDX models reveals patterns of neuroblastoma chemoresistance. *Sci Adv*. 2024;8(43):eabq4617. doi:10.1126/sciadv.abq4617

48. Zhu S, Lee JS, Guo F, et al. Activated ALK collaborates with MYCN in neuroblastoma pathogenesis. *Cancer Cell*. 2012;21(3):362-373. doi:10.1016/j.ccr.2012.02.010

49. Zimmerman MW, Liu Y, He S, et al. MYC Drives a Subset of High-Risk Pediatric Neuroblastomas and Is Activated  through Mechanisms Including Enhancer Hijacking and Focal Enhancer Amplification. *Cancer Discov*. 2018;8(3):320-335. doi:10.1158/2159-8290.CD-17-0993

50. Zhu S, Zhang X, Weichert-Leahey N, et al. LMO1 Synergizes with MYCN to Promote Neuroblastoma Initiation and Metastasis. *Cancer Cell*. 2017;32(3):310-323.e5. doi:10.1016/j.ccell.2017.08.002

51. He S, Mansour MR, Zimmerman MW, et al. Synergy between loss of NF1 and overexpression of MYCN in neuroblastoma is  mediated by the GAP-related domain. *Elife*. 2016;5. doi:10.7554/eLife.14713

52. Zhang X, Dong Z, Zhang C, et al. Critical Role for GAB2 in Neuroblastoma Pathogenesis through the Promotion of  SHP2/MYCN Cooperation. *Cell Rep*. 2017;18(12):2932-2942. doi:10.1016/j.celrep.2017.02.065

53. Tao T, Shi H, Mariani L, et al. LIN28B regulates transcription and potentiates MYCN-induced neuroblastoma through  binding to ZNF143 at target gene promotors. *Proc Natl Acad Sci U S A*. 2020;117(28):16516-16526. doi:10.1073/pnas.1922692117

54. Ribatti D, Tamma R. The chick embryo chorioallantoic membrane as an in vivo experimental model to  study human neuroblastoma. *J Cell Physiol*. 2018;234(1):152-157. doi:10.1002/jcp.26773
